# Emerging roles for diguanylate cyclase during the evolution of soma in dictyostelia

**DOI:** 10.1186/s12862-023-02169-z

**Published:** 2023-10-06

**Authors:** Yoshinori Kawabe, Qingyou Du, Takaaki B. Narita, Craig Bell, Christina Schilde, Koryu Kin, Pauline Schaap

**Affiliations:** 1 Molecular Cell and Developmental Biology, School of Life Sciences, University of Dundee, Dundee, DD15EH UK; 2 Department of Life Science, Faculty of Advanced Engineering, Chiba Institute of Technology, Chiba, 275-0016 Japan; 3 West of Scotland Innovation Hub, NHS Greater Glasgow and Clyde, Queen Elizabeth University Hospital, Glasgow, G514LB UK; 4 D’Arcy Thompson Unit, School of Life Sciences, University of Dundee, Dundee, DD14HN UK; 5 Institut de Biologia Evolutiva (CSIC-Universitat Pompeu Fabra), Barcelona, 08003 Spain

**Keywords:** Dictyostelid social amoebas, Evolution of soma, Diguanylate cyclase, Adenylate cyclase A, ammonia transporter C, Prolyl 4-hydroxylase, Polyketide synthase steely B

## Abstract

**Background:**

Cyclic di-guanylate (c-di-GMP), synthesized by diguanylate cyclase, is a major second messenger in prokaryotes, where it triggers biofilm formation. The dictyostelid social amoebas acquired diguanylate cyclase (*dgcA*) by horizontal gene transfer. *Dictyostelium discoideum* (*Ddis*) in taxon group 4 uses c-di-GMP as a secreted signal to induce differentiation of stalk cells, the ancestral somatic cell type that supports the propagating spores. We here investigated how this role for c-di-GMP evolved in Dictyostelia by exploring *dgcA* function in the group 2 species *Polysphondylium pallidum* (*Ppal*) and in *Polysphondylium violaceum* (*Pvio*), which resides in a small sister clade to group 4.

**Results:**

Similar to *Ddis*, *dgcA* is upregulated after aggregation in *Ppal* and *Pvio* and predominantly expressed in the anterior region and stalks of emerging fruiting bodies. *DgcA* null mutants in *Ppal* and *Pvio* made fruiting bodies with very long and thin stalks and only few spores and showed delayed aggregation and larger aggregates, respectively. *Ddis dgcAˉ* cells cannot form stalks at all, but showed no aggregation defects. The long, thin stalks of *Ppal* and *Pvio dgcAˉ* mutants were also observed in *acaAˉ* mutants in these species. *AcaA* encodes adenylate cyclase A, which mediates the effects of c-di-GMP on stalk induction in *Ddis.* Other factors that promote stalk formation in *Ddis* are DIF-1, produced by the polyketide synthase StlB, low ammonia, facilitated by the ammonia transporter AmtC, and high oxygen, detected by the oxygen sensor PhyA (prolyl 4-hydroxylase). We deleted the single *stlB*, *amtC* and *phyA* genes in *Pvio* wild-type and *dgcAˉ* cells. Neither of these interventions affected stalk formation in *Pvio* wild-type and not or very mildly exacerbated the long thin stalk phenotype of *Pvio dgcAˉ* cells.

**Conclusions:**

The study reveals a novel role for c-di-GMP in aggregation, while the reduced spore number in *Pvio* and *Ppal dgcAˉ* is likely an indirect effect, due to depletion of the cell pool by the extended stalk formation. The results indicate that in addition to c-di-GMP, Dictyostelia ancestrally used an as yet unknown factor for induction of stalk formation. The activation of AcaA by c-di-GMP is likely conserved throughout Dictyostelia.

**Supplementary Information:**

The online version contains supplementary material available at 10.1186/s12862-023-02169-z.

## Background

Early transitions to multicellularity likely had several causes such as defence against predation [1], improved movement or feeding by co-ordinated beating of flagella [2] and the ability to form photosynthetic mats [3]. In early multicellular forms, cells were likely still phenotypically identical. However, in most of their descendants, specialization occurred into cells that propagate the organism and others that support propagation by providing structural support, enhancing motility and facilitating nutrient uptake and processing. In modern organisms, such somatic cells are now present in much larger numbers and functional varieties than the propagating cells.

We are interested in understanding how somatic cell type specialization evolved, using the relatively simple dictyostelid social amoebas as a model organism. The Dictyostelia are unicellular when feeding, but aggregate to form multicellular fruiting structures with dormant spores when their bacterial food source is exhausted. They constitute one of two clades within the otherwise unicellular Amoebozoa (the other being the Copromyxa) that developed this type of aggregative multicellularity [4]. The Dictyostelia are themselves subdivided into two branches that each contain two major groups [5]. The clade of Acytostelids within group 2 forms structures that consist only of propagating spores, supported by a cellulose tube, while the other species in groups 1, 2 and 3 have evolved a single somatic cell type that is organised into a linear array enclosed in a cellulose tube to form a cellular stalk. The group 4 Dictyostelids, which contain the model *Dictyostelium discoideum*, underwent major phenotypic innovations, amongst which the specialization of three more somatic cell types that make up a basal disc to support the stalk and upper and lower cups that lift and cradle the spore head [6].

The spores of Dictyostelia are ancestrally derived from the walled dormant cysts that are formed by unicellular Amoebozoa in response to starvation or other forms of stress. Stress induces an increase in intracellular cAMP in tested Amoebozoa, which acts on cAMP dependent protein kinase (PKA) to activate encystation [7–10]. In multicellular fruiting bodies of *D. discoideum*, cAMP acting on PKA activates the maturation of prespore and prestalk cells into spores and stalk cells respectively [11, 12]. Prespore cells start to differentiate just after aggregation by prefabricating part of the spore wall in Golgi-derived vesicles. This process is induced combinatorially by extracellular cAMP acting on cAMP receptors (cARs) and intracellular cAMP acting on PKA [13–15]. Prestalk and stalk cell differentiation were previously proposed to be induced by the polyketide DIF-1 [16, 17], but DIF-1 later proved to be mainly responsible for the differentiation of the basal disc [18]. Stalk formation is however induced by c-di-GMP [19], which acts on adenylate cyclase A (ACA) to increase cAMP levels and activate PKA [20]. C-di-GMP is an important second messenger in bacteria [21], and diguanylate cyclase, the enzyme that synthesizes c-di-GMP, entered the last common ancestor to Dictyostelia by horizontal gene transfer [22].

Evolutionary comparative studies indicate that the roles of extracellular and intracellular cAMP acting on cARs and PKA, respectively, are conserved in at least the group 2 species *Polysphondylium pallidum (Ppal)* [9, 23, 24], while DIF-1 was detected in other group 4 species and in *Polysphondylium violaceum (Pvio)*, a sister species to group 4, but not in the more distantly related group 3 [25]. Strikingly, DIF-1 inhibits rather than promotes stalk cell differentiation in *P. violaceum* [26]. Here we investigate functional conservation of c-di-GMP as inducer of the first somatic cell type in Dictyostelia by deleting the diguanylate cyclase gene, *dgcA*, across non-group 4 species. We found that both in *Pvio* and *Ppal*, loss of *dgcA* resulted in fruiting bodies with very thin and long stalks, accompanied by a large reduction in sporulation efficiency. We explored whether other positive regulators of *Ddis* stalk cell differentiation such a DIF-1, high oxygen levels and loss of ammonia showed overlapping roles with c-di-GMP in proper stalk formation, by generating double knock-outs of *dgcA* with the synthetic enzyme, sensor and export facilitator for these factors, respectively.

## Results

### Expression pattern of *P. pallidum dgcA*

**Fig. 1:**
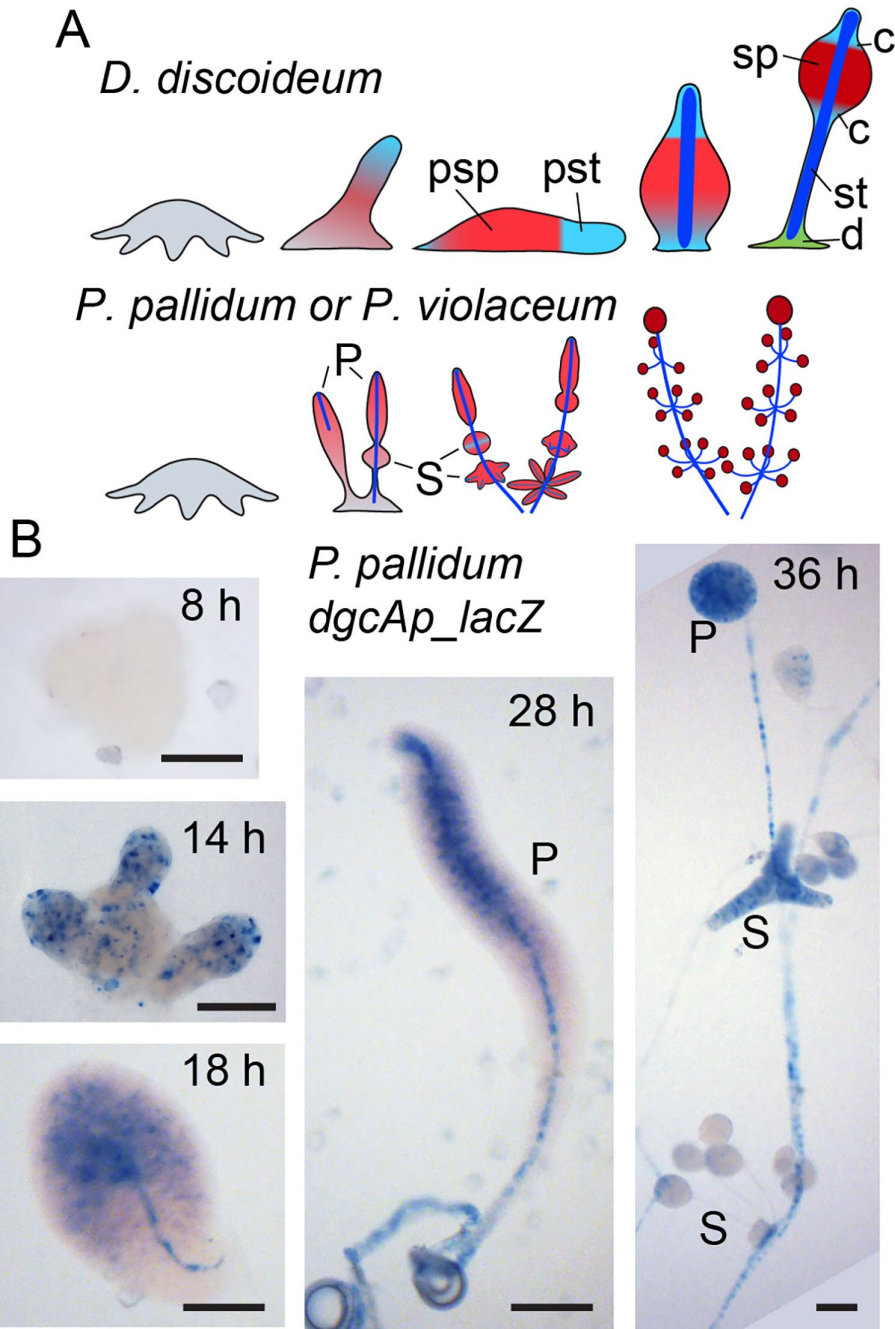
**Expression pattern of the*P. pallidum dgcA *gene**. (**A**) *Fruiting body morphogenesis in D. discoideum and the Polysphondylia.* Pst: prestalk cells; psp: prespore cells; sp: spores; st:stalk; d: basal disc; c: upper and lower cup; P; primary sorogen; S: secondary sorogen. (**B**) *DgcA expression in P. pallidum.* A gene fusion of *lacZ* and 2.5 kb of the *Ppal dgcA* intergenic region was transformed into *Ppal* cells and plated for development. Structures that had formed at the indicated time points were fixed and stained with X-gal. Bar: 100 μm. Representative images from at least three individual experiments

*DgcA* is present as a single copy gene in most Dictyostelia, but underwent amplification to a total of 13 genes in the group 1 species *D. fasciculatum* (see Additional File 1, Figure S1). In *D. discoideum, dgcA* promoter activity was predominantly found in prestalk and stalk cells [19]. Comparative transcriptomics shows that in all four taxon groups *dgcA* is upregulated in late development in stalk cells, but that there is also some expression in growing cells and for *D.discoideum* (group 4) in cup cells and *D. lacteum* (group 3) in spores (Figure S1). To gain initial insight into a role for DgcA across Dictyostelia, we examined the *dgcA* expression pattern of the group 2 species *P. pallidum (Ppal)* in more detail by fusing the *Ppal dgcA* promoter to the *LacZ* reporter gene. Figure 1 shows that *dgcA* becomes expressed after aggregation in the upper half of the emerging primary sorogens and in the stalk. The secondary whorls of sorogens that are formed from the rear of the primary sorogen mostly express *dgcA* throughout as do the primary spore heads and the stalks. Note, that like most group 1–3 species, *Ppal* has almost no prestalk region, since its sorogens largely consist of prespore cells, which transdifferentiate into stalk cells when they approach the tip [27]. The extended pattern of *dgcA* expression in *Ppal* is different from *Ddis*, where *dgcA* expression is mostly restricted to prestalk cells and some scattered cells throughout the prespore region [19].

### *Loss of Ppal dgcA* progressively delays aggregation, side branching and sporulation

**Fig. 2:**
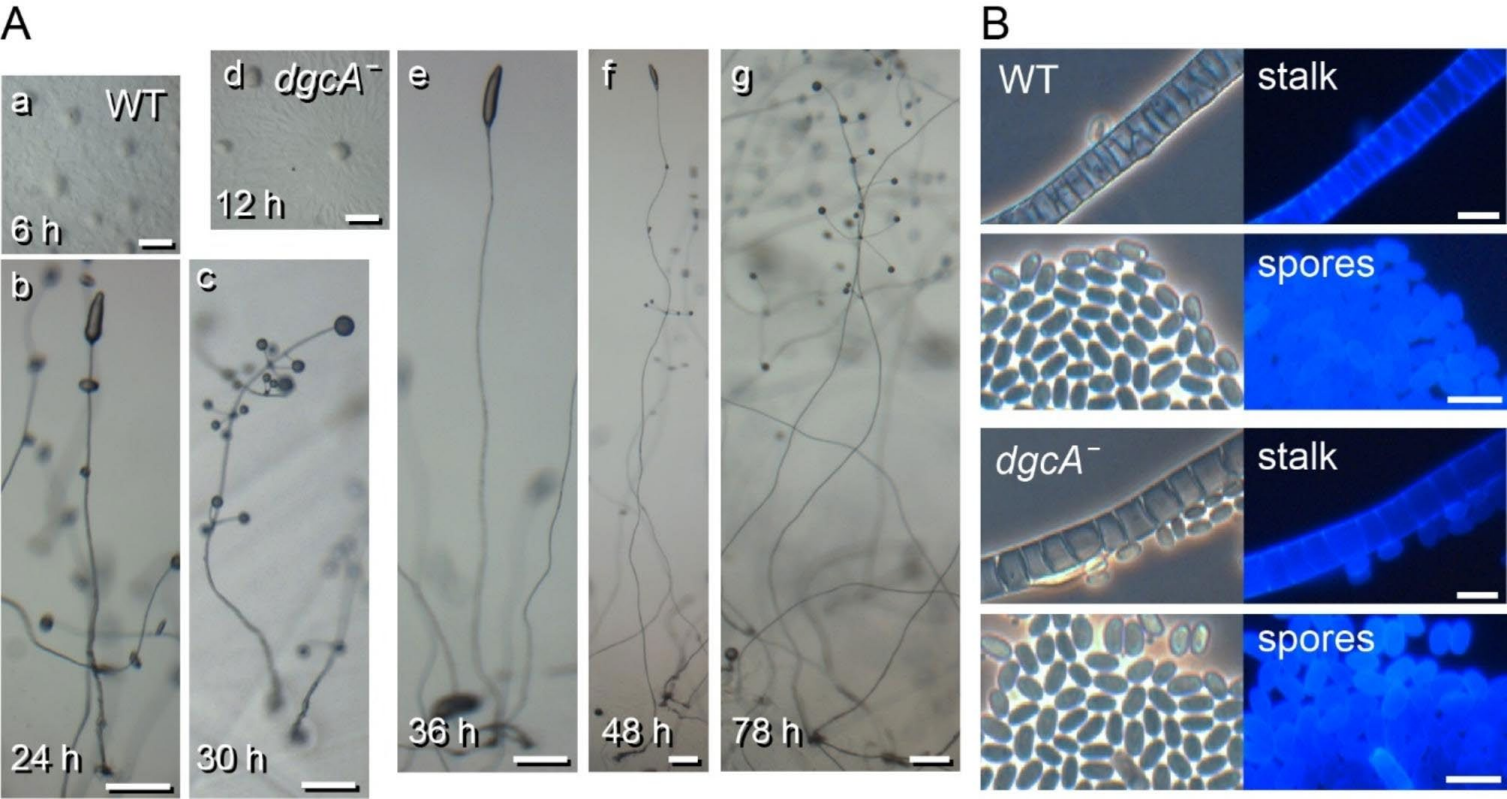
**Development and differentiation of the*P. pallidum dgcAˉ*mutant**. *Ppal* wild type and *dgcAˉ* cells were incubated at 22 °C on NN agar at 10^6^ cells/cm^2^. (**A**) *Development*. a-c wildtype cells. a, aggregation; b, primary sorogen and branch formation and c, fruiting bodies. d-g *dgcaˉ* cells. d, aggregation; e, primary sorogen; f, primary sorogen and branch formation; g, fruiting bodies. Scale bars: 0.25 mm. The images are representative of phenotypes observed from at least four separate experiments. (**B**) *Cell differentiation*. The fruiting bodies were stained with 0.001% Calcofluor and photographed under phase contrast (left) and epifluorescence (right). Scale bar: 10 μm

To assess the function of DgcA in *Ppal*, a *dgcA* lesion was introduced by homologous recombination (see Additional File 1, Figure S2). The *dgcAˉ* cells aggregated 3 to 6 h later than wild-type *Ppal* and initially formed normal primary sorogens, but the formation of secondary sorogens, which split off from the rear of the primary sorogen and give rise to the side branches, was much delayed. The primary stalk continued to extend for about 40 h after the wild-type fruiting bodies had already matured and as a result, very tall fruiting bodies were formed with side branches only at the upper stalk (Fig. 2A). Staining of the *dgcA*ˉ stalk and spore cells with the cellulose dye Calcofluor showed that *dgcA*ˉ formed a normal stalk and elliptical spores, encapsulated in cellulose walls (Fig. 2B).

Evidently, *Ppal* DgcA is not essential for formation of the primary stalk, as is the case in *Ddis*, although it is essential for timely aggregation, secondary sorogen formation and spore maturation.

### *P. violaceum dgcA* is expressed in tip and stalk cells

*Ppal* was until recently the only non-group 4 species that could be transformed, limiting our ability to investigate the role of DgcA more broadly. However, we recently developed transformation and gene knock-out procedures for *P. violaceum (Pvio)* [26], which resides in a small sister clade to group 4 (figure S1) and is therefore more closely related to *Ddis* than *Ppal*. Genome and cell-type specific transcriptome data are also available for this strain [26] and a developmental transcriptome was recently prepared (Supplementary_Dataset_File1_ Pvio_RNAseq.xlsx).

**Fig. 3:**
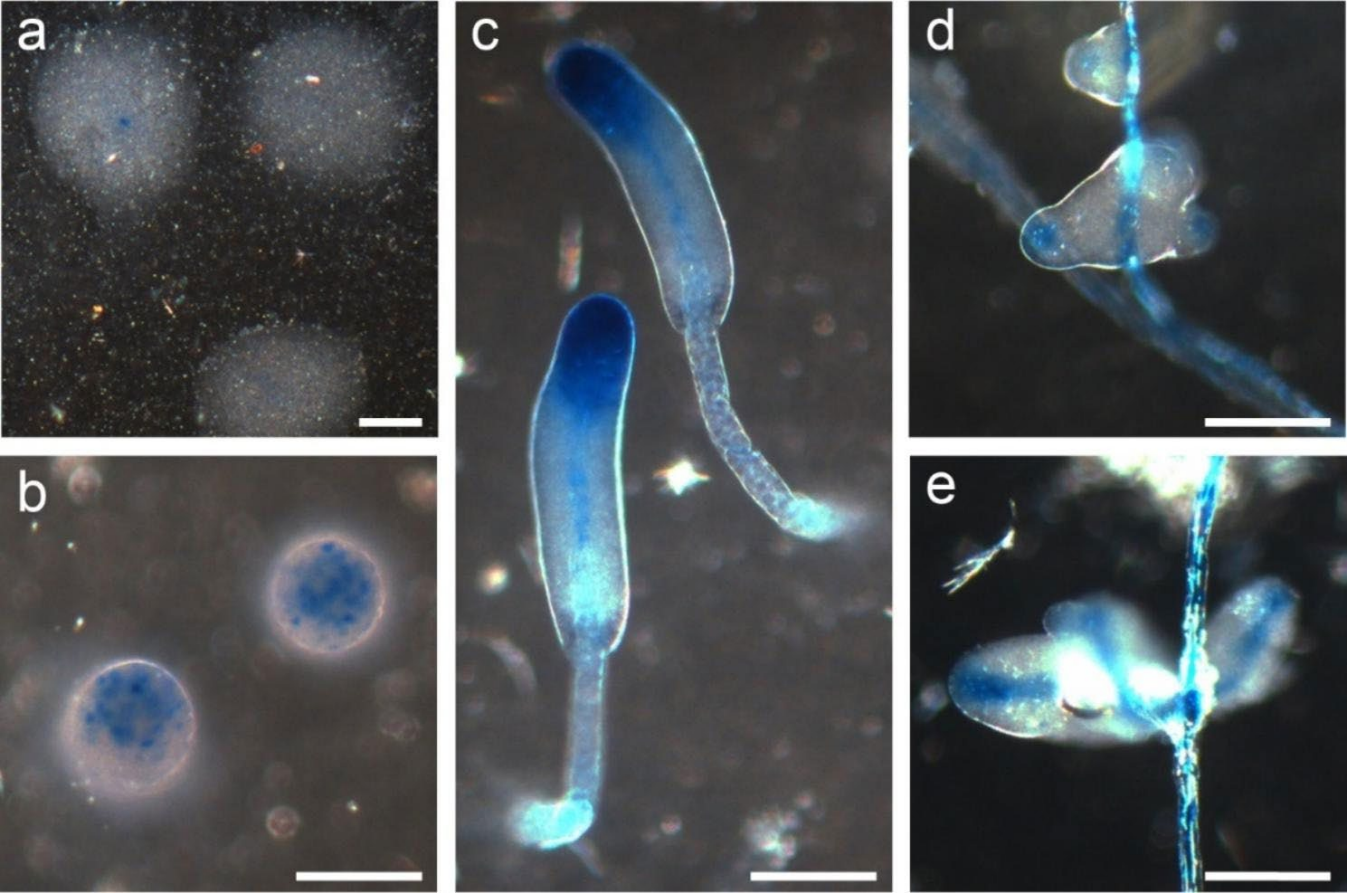
**Expression pattern of*Pvio dgcA***. The 2.8 kb 5’-intergenic region of *Pvio dgcA* was fused to *lacZ* and expressed in *Pvio* wild-type. The cells were plated for development until the indicated stages were reached, and then fixed and stained with X-gal. a, early aggregate; b, late aggregate; c, primary sorogen; d, tip formation in segregating whorls; e, secondary sorogens. Scale bars: 0.1 mm. Representative images from two different experiments of two separately developed clones are shown

The transcriptome data show that, similar to *Ddis* and *Ppal dgcA*, *Pvio dgcA* is upregulated after aggregation, but also shows some expression in growing cells (Fig. S1). To gain information on the spatial expression pattern of *dgcA*, we transformed *Pvio* cells with a *Pvio dgcA* promoter *LacZ* fusion construct. In aggregates, *dgcA* is first expressed in a few cells at the aggregation centre and then becomes expressed more strongly at the tip of the emerging sorogen (Fig. 3). When the sorogen matures, *dgcA* expression is confined to the stalk and to the top third of the sorogen. In secondary sorogens, *dgcA* is also first expressed at the tip and later in the stalk. With respect to its prestalk and stalk specificity, this expression pattern is more similar to that of *Ddis dgcA* [19] than that of *Ppal dgcA*, which is expressed more posteriorly in the primary and secondary sorogens (Fig. 1).

### ***Pvio dgcAˉ*** fruiting bodies have long thin stalks and few spores

**Fig. 4:**
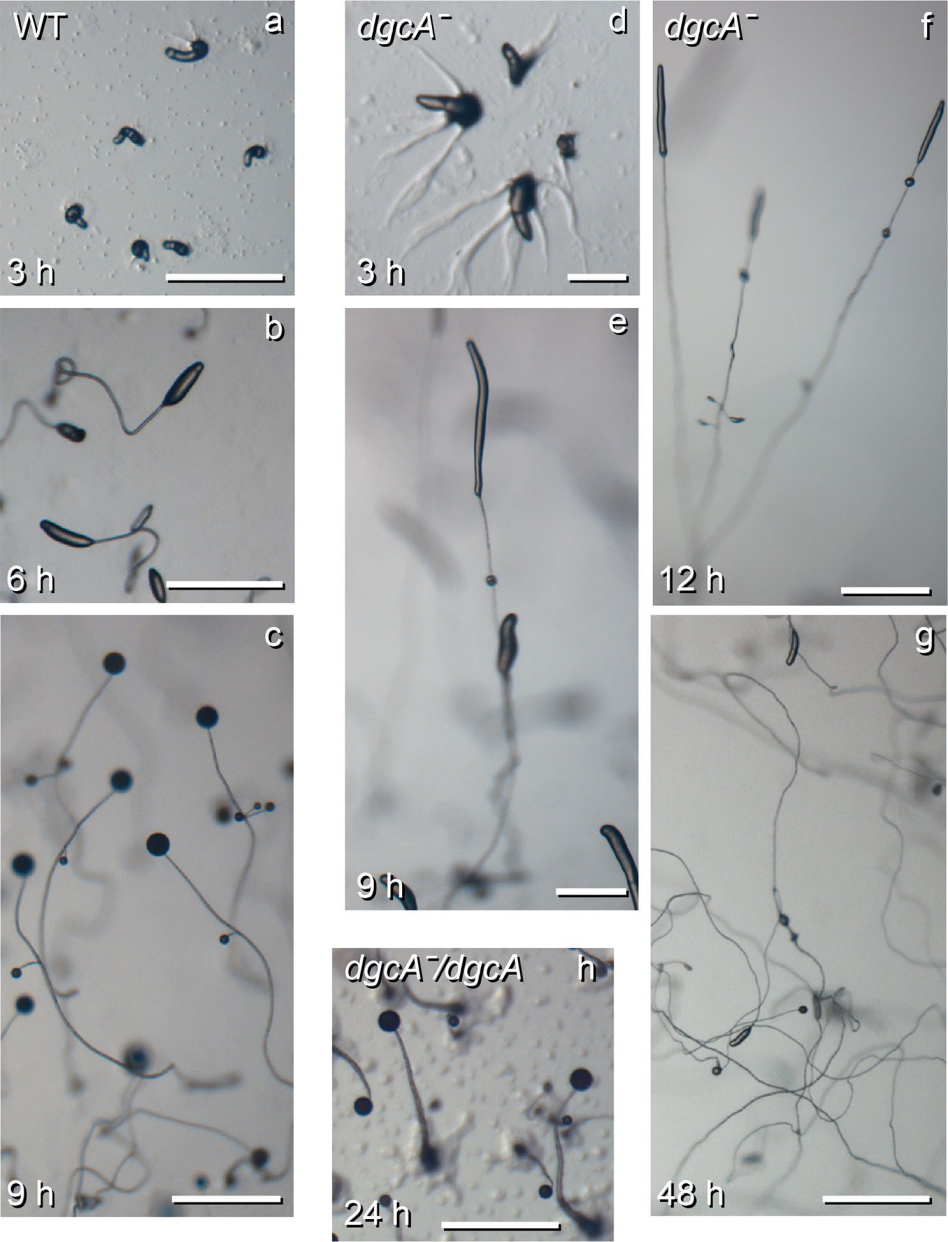
**Development of the*Pvio dgcAˉ*mutant**. *Pvio* wild type, *dgcAˉ* and *dgcAˉ* cells complemented with *dgcA* were distributed at 10^6^ cells/cm^2^ on KK2 agar, incubated overnight at 4 °C and for the indicated periods at 22^o^C. Structures were imaged at comparable developmental stages, which where reached at different time periods for the three strains a-c: wild-type *Pvio. a*, late aggregation and tip formation; b, primary sorogens; c, mature fruiting bodies. d-g: *Pvio dgcAˉ*. d, late aggregation and tip formation; e, separation of first whorl; f, side branch formation; g, mature fruiting bodies and some sorogens still forming stalk. Representative images from at least two independent experiments comprising two different knock-out clones are shown. h: mature fruiting bodies of *dgcAˉ/dgcA* cells. Scale bars: 0.5 mm. Representative images from three independent experiments are shown

To generate a *dgcA* lesion in *Pvio*, cells were transformed with a construct in which part of the *dgcA* sequence was replaced with the G418 resistance cassette. All observed G418 resistant clones showed the same abnormal fruiting bodies and Southern blot analysis indicated that homologous recombination with *dgcA* had occurred in three of such clones (Figure S3). Such high efficiency of gene knock-out was also observed in earlier experiments [26]. To synchronize development, *Pvio dgcAˉ* and wild-type cells were starved on KK2 agar at 4ºC overnight and then transferred to 22ºC. Both wild-type and *dgcAˉ* cells aggregated after 3 h at 22ºC. However, the *dgcAˉ* cells made larger aggregates and then formed abnormal fruiting bodies with very long and thin stalks (Fig. 4). Most wild-type cells completed fruiting body formation within 9 h, but the primary stalks of *Pvio dgcA*^−^ continued to extend for several days and finally formed a small spore head. Both *Pvio* wild-type and *dgcAˉ* only formed sparse whorls of side branches.

**Fig. 5:**
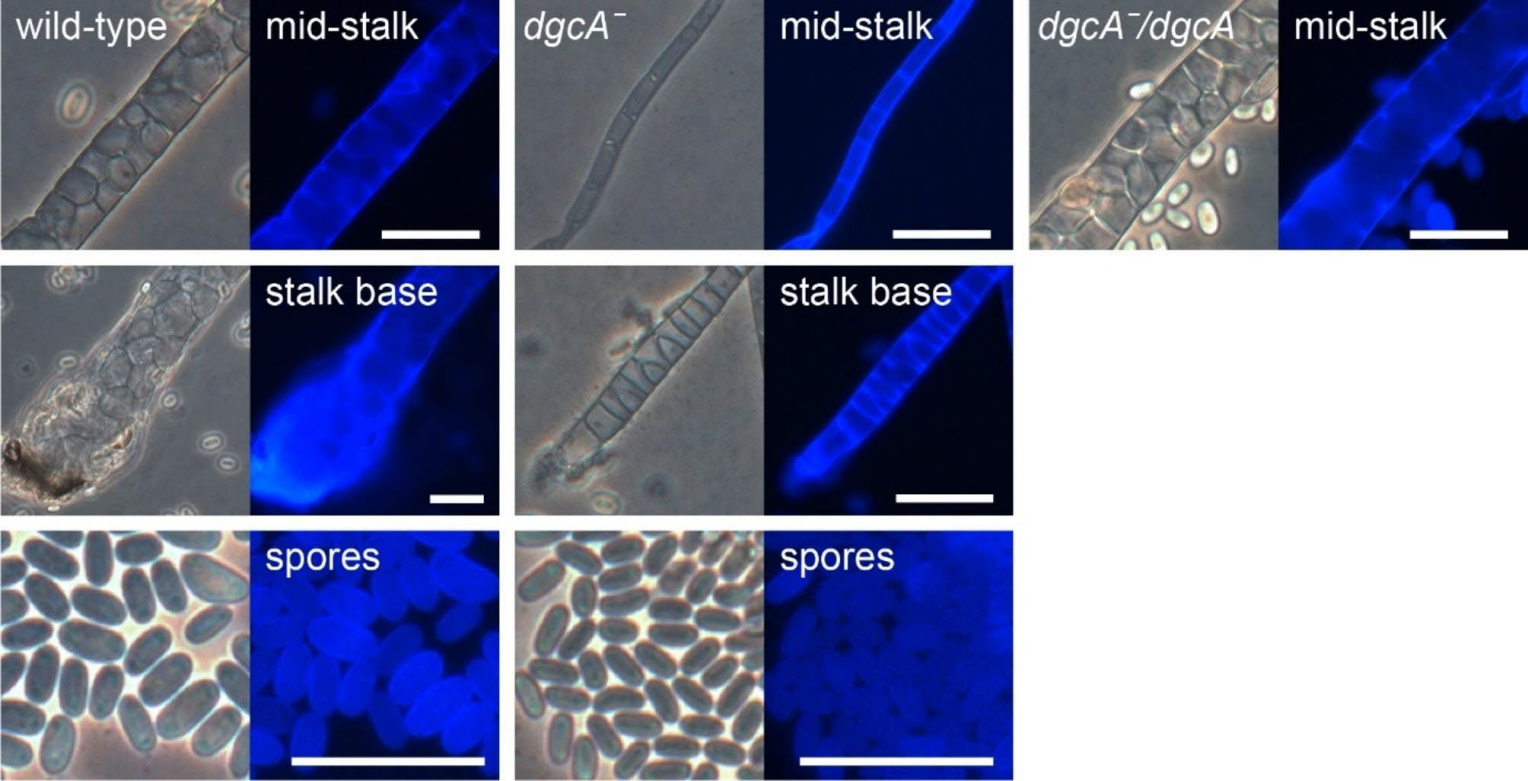
**Stalks and spores in*Pvio dgcAˉ*mutants**. *Pvio* wild type, *dgcAˉ* and *dgcAˉ/dgcA cells* were developed into fruiting bodies as outlined for Fig. 4. The structures were stained with 0.001% Calcofluor and photographed under phase contrast (left) and epifluorescence (right). The top and middle rows shows stalks imaged about half-way up and at their base, respectively, while the bottom rows show the spores. Images are representative of two experiments performed with two different *dgcAˉ* clones and three experiments of the *dgcAˉ/dgcA* cell line. Scale bar: 20 μm

Staining with the cellulose dye Calcofluor revealed that similar to *Ppal dgcAˉ*, but unlike *Ddis dgcAˉ*, *Pvio dgcAˉ* cells formed mature spore and stalk cells (Fig. 5). However, the stalk, which is in wild-type *Pvio* 2 or 3 cells wide, consisted of only a single tier of elongated cells in the *Pvio dgcAˉ* mutant. This difference was particularly evident at the base of the stalk, which is several cells wide in wild-type, but only 1 or 2 cells wide in the *dgcAˉ* mutant. The spores in the terminal *Pvio dgcAˉ* spore head showed a normal elliptical shape and Calcofluor positive cell wall, but were markedly smaller than those of wild-type *Pvio*. This is likely due to the fact that they took several days to mature, which in all Dictyostelia occurs in the absence of nutrition.

To confirm that the *Pvio dgcAˉ* phenotype was caused by loss of *dgcA*, a *dgcA* expression vector, which contains the promoter, coding sequence and terminator of *Pvio dgcA* was transformed into the *dgcAˉ* mutant. Similar to wild-type the *dgcAˉ/dgcA* cells formed robust fruiting bodies within 24 h, with stalks that were several cells wide (Figs. 4h and 5). This indicates that the thin stalk and delayed development of the *dgcAˉ* mutant was caused by loss of DgcA.

### Spore, prespore and stalk cell differentiation in ***dgcAˉ*** mutants

**Fig. 6:**
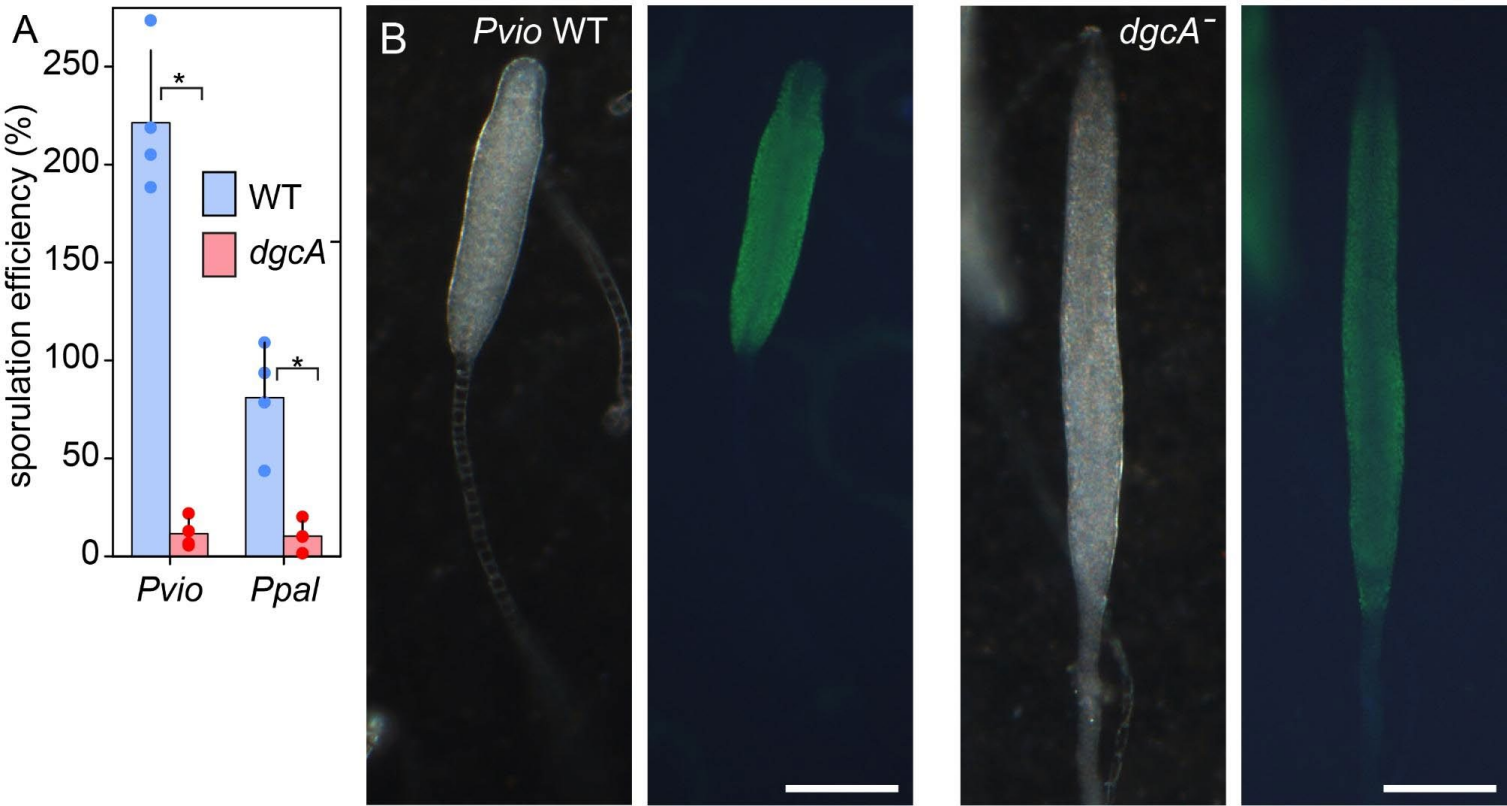
**Spore and prespore differentiation in*Pvio dgcAˉ***. (**A**) Sporulation efficiency. *Ppal* and *Pvio* wild-type and *dgcAˉ* cells were developed into mature fruiting bodies on 2 × 2 cm nitrocellulose filters at 4 × 10^6^ cells/filter. The filters were then vigorously shaken in 4 ml 0.1% Triton X-100, spores were counted and the percentage of spores relative to the number of plated cells was determined. Experiment averages as well as means and SD of four experiments, assayed with two filters each, are presented. *: significantly different at P < 10^− 5^ as determined by a t-test. (**B**) *Pvio* wild-type and *dgcAˉ* primary sorogens were fixed in methanol and stained with rabbit antispore antibodies [27] and FITC-conjugated goat-anti-rabbit-IgG. Structures were photographed under dark field (left) and epifluorescence (right). Images representative of two experiments are shown. Bar: 0.2 mm

Both *Ppal* and *Pvio dgcAˉ* sorogens delay spore maturation and continue to produce thin stalks for an extended period (Figs. 2 and 4) To assess whether the excessive stalk formation reduces the number of cells available to form spores, we measured sporulation efficiency i.e. the number of spores formed from a known number of cells for the *dgcAˉ* and wild-type cells of both species. For wild-type *Ppal*, 81% of plated cells differentiated into spores, but for wild-type *Pvio* this was 221% (Fig. 6A). Such amplification was also observed in *Ddis* [28] and likely signifies that the cells still go through one or two rounds of cell division after being deprived of food. Compared to wild-type, sporulation efficiency was 87% reduced in *Ppal dgcAˉ* and 95% in *Pvio dgcAˉ.* Some *Pvio dgcAˉ* aggregates never lifted off the filters at all, which likely exacerbates its poor sporulation efficiency.

Spore differentiation occurs in two stages. Shortly after aggregation most cells in the emerging sorogen start to prefabricate spore wall materials in Golgi-derived vesicles [29]. Once the sorogen has risen some distance on the emerging stalk, the vesicles are exocytosed and the spore wall is fully synthesized. To investigate which stage of spore differentiation is perturbed in the *dgcAˉ* mutants, we stained early to mid sorogens with an antibody raised against a mixture of *Ddis* and *Ppal* spores that reacts to prespore vesicles and spores in all *Dictyostelium* species [27]. Figure 6B shows that both *Pvio* wild-type and *dgcAˉ* sorogens stained with anti-spore antibodies. As observed previously [30], the *Pvio* wild-type sorogens showed staining almost up to the tip, while the newly differentiating and more mature stalk cells were devoid of staining. This was also the case for the *dgcAˉ* sorogens, indicating that most cells did differentiate into prespore cells.

### Effect of *stlB* deletion in *Pvio dgcAˉ* cells

While *Ddis dgcAˉ* cells do not form a stalk at all, both *Pvio dgcAˉ* still forms thin stalks, suggesting that here c-di-GMP is not absolutely essential for stalk formation. In addition to c-di-GMP, the chlorinated polyketide DIF-1 also induces differentiation of stalk-like cells in *Ddis* [16]. Here loss of StlB, which synthesizes the polyketide backbone of DIF-1, results in weak stalks and loss of the basal disc that consists of stalk-like cells [18]. In *Pvio*, deletion of *stlB* resulted in formation of misshapen and thicker lower stalks and reduced prespore gene expression [26].

**Fig. 7:**
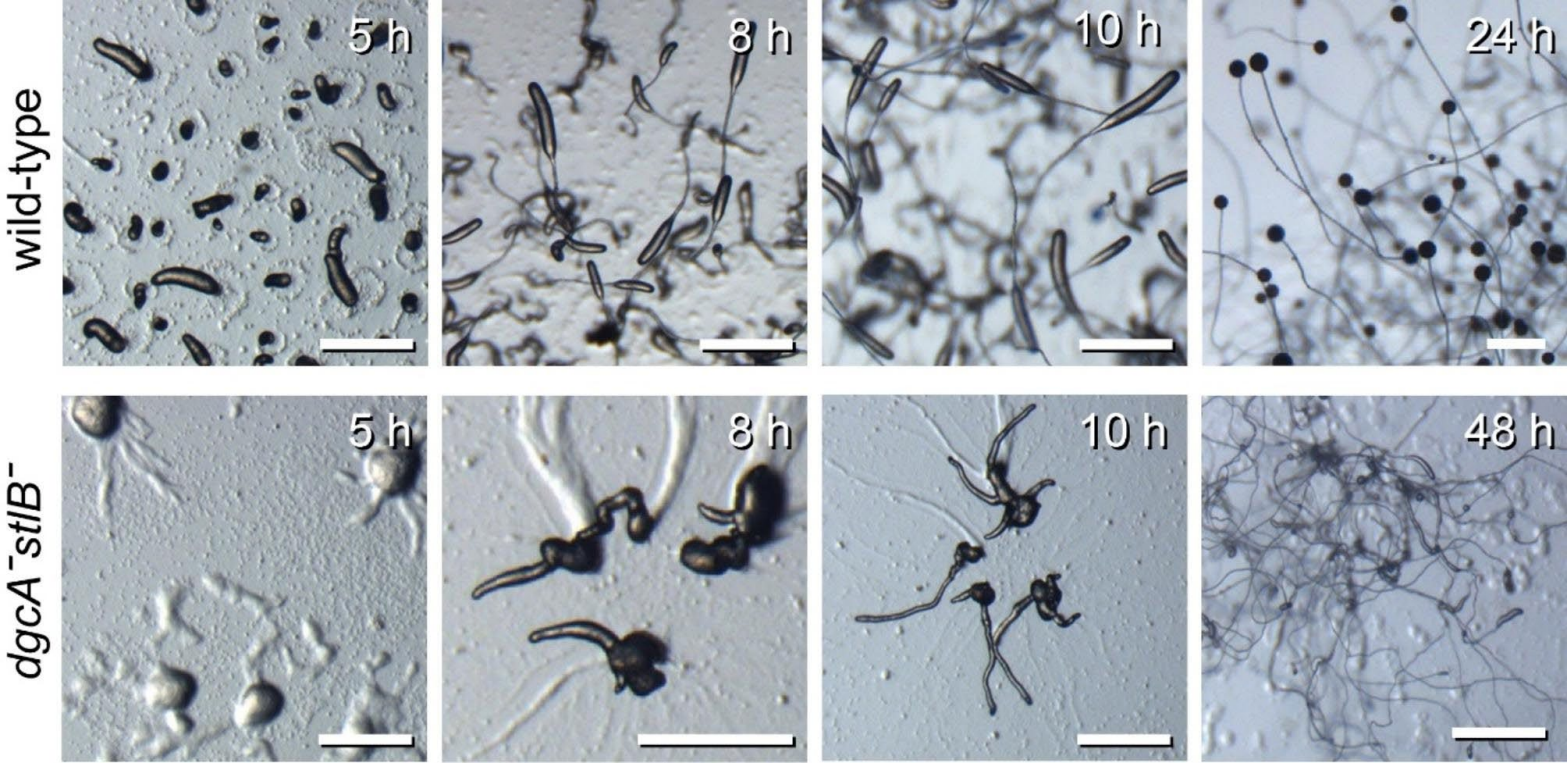
**Phenotype of the*Pvio dgcAˉstlBˉ*mutant**. Wild-type *Pvio* and a *Pvio dgcAˉstlBˉ* double mutant were plated on KK2 agar, starved overnight at 4ºC and for the indicated periods at 22ºC and photographed. Scale bar: 1 mm. Representative images from three experiments on four *dgcAˉstlBˉ* clones.

To assess whether the residual stalk cell differentiation in *Pvio dgcAˉ* could be due to DIF-1 synthesis, we deleted *stlB* in a *Pvio dgcAˉ* mutant, from which the loxP-neo^R^ selection cassette was removed by transformation with cre-recombinase. Similar to the *Pvio dgcAˉ* mutant, the *dgcAˉstlBˉ* formed large aggregates, which then split up to form many irregularly shaped sorogens with initially delayed uplift of the main cell mass (Fig. 7). However after uplift the main mass continued to produce very long and thin stalks and ultimately only a very small spore head. The phenotype of *dgcAˉstlBˉ* mutant thus appeared to combine the phenotypes of the *dgcAˉ* and *stlBˉ* mutants, with both deletions probably contributing to the low sporulation efficiency. However, because a stalk was still formed in the *dgcAˉstlBˉ* mutant, it is unlikely that StlB and DgcA have overlapping roles in stalk induction.

### Effects of *amtC* and *phyA* deletion in *Pvio* wild-type and *dgcAˉ cells*

In *Ddis* c-di-GMP triggers stalk formation by activating cAMP synthesis by the adenylate cyclase ACA, which is preferentially expressed at the sorogen tip. cAMP then activates PKA which causes the transition of prestalk into stalk cells [20]. The catabolite ammonia, which is produced by protein degradation through autophagy in the starving cells, is a negative regulator of stalk cell differentiation in *Ddis* [31, 32]. Ammonia activates the histidine kinase DhkC, which by phosphorylating the cAMP phosphodiesterase RegA, lowers cAMP levels, preventing activation of PKA [33]. The ammonia transporter AmtC, which is expressed at the sorogen tip and in prespore cells, facilitates local loss of ammonia, allowing the *Ddis* stalk and thereby the fruiting body to form [34]. The other *Ddis* transporters AmtB and AmtA also export ammonia [35], but have either no known role (AmtB) or act as an ammonia sensor (AmtA), antagonizing the role of AmtC [36].

**Fig. 8:**
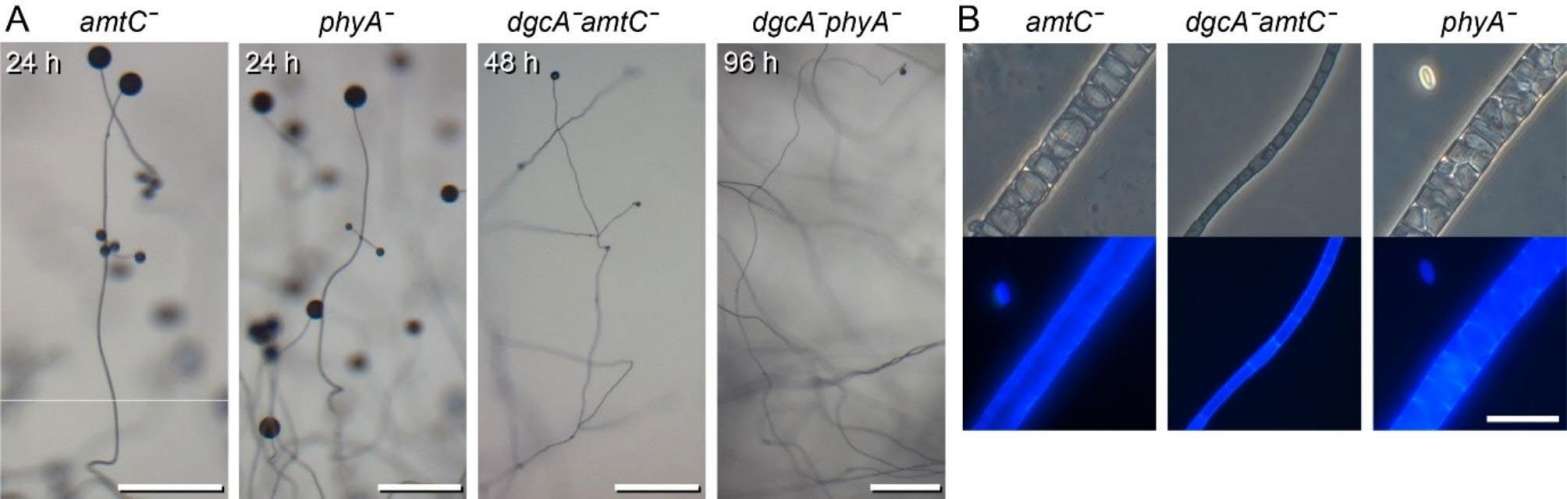
**Phenotype of the*Pvio amtCˉ***, ***phyAˉ, dgcAˉamtCˉ and dgcAˉphyAˉ*mutants**. (**A**) *Fruiting bodies*. *AmtCˉ*, *phyAˉ*, *dgcAˉamtCˉ* and *dgcAˉphyAˉ* cells were incubated overnight at 4 °C on KK_2_ agar and then transferred to 22^o^C for the indicated time periods until mature fruiting bodies had formed. Scale bars: 0.5 mm. (**B**) *Stalk cells.* The fruiting bodies were stained with 0.001% Calcofluor and stalks were photographed under phase contrast (top) and epifluorescence (bottom). Scale bars: 10 μm. Representative images from at least two individual experiments

BLASTp search and phylogenetic inference identified Pvio_g4319 as an ortholog of *Ddis amtC*, with the other *Ddis* ammonia transporters being more diverged (Figure S1). To test whether loss of ammonia through AmtC could be responsible for residual stalk cell diffentiation in *Pvio*, we deleted *amtC* in *Pvio* wild-type and *dgcAˉ* cells, for the latter using the G418 sensitive *dgcAˉ* clone, generated as described above. The *amtCˉ* mutant showed similar developmental progression and fruiting body morphology as wild-type *Pvio*, while the phenotype of the *dgcAˉamtCˉ* double mutant was the same as that of *dgcAˉ*, with a thin and long stalk and very small spore heads (Fig. 8). These data suggest that AmtC has no essential role in regulating stalk cell differentiation in *Pvio.*

Ample oxygen availability is another factor that is required for stalk cell differentiation. Below 10% oxygen *Ddis* wild-type cells still form migrating slugs but never form fruiting bodies [37], as is also the case for *Ddis dgcAˉ* cells [19]. Oxygen is sensed by the prolyl 4-hydroxylase PhyA, which hydroxylates proline 143 of Skp1A or Skp1B, each a subunit of the SCF (Skp, Cullin and F-box) complex E3-ubiquitin ligase, which after further modification of the hydroxylated proline causes ubiquitination of an as yet unknown target protein and its degradation by the proteasome. *Ddis phyAˉ* cells also form slugs but cannot form fruiting bodies [38, 39]. To investigate whether this oxygen signalling pathway might control stalk cell differentiation in the *Pvio dgcAˉ* mutant, we deleted the single *Pvio phyA* gene in both wild-type and *dgcAˉ* cells (Figures S1 and S6).

The *Pvio phyAˉ* mutant showed normal stalk and fruiting body formation, while the phenotype of the double *dgcAˉphyAˉ* mutant was similar to that of *Pvio dgcAˉ* with long thin stalks and very small spore heads (Fig. 8). Evidently, oxygen sensing by *phyA* does not play a major role in fruiting body development in *Pvio*.

## Discussion

### *Ppal* and *Pvio dgcAˉ* mutants form long and thin stalks and few spores

Cyclic-di-GMP is an essential inducer of stalk formation in the group 4 species *Ddis*, where loss of its synthetic enzyme DgcA results in prolonged slug migration and failure to erect a fruiting body [19]. To assess the evolutionary history for this role of c-di-GMP, we deleted the *dgcA* from *Ppal* in group 2 and from *Pvio* in the closest sister clade to group 4 and examined its expression pattern in both species. Similar to *Ddis*, *dgcA* expression is upregulated after aggregation in *Ppal* and *Pvio* (Figs. S1, 1 and 3), and mainly occurs in the anterior region of sorogens and in the stalk. However, in *Ppal* expression extends into the prespore region and is also found in mature spores.

Loss of *dgcA* in *Ppal* delayed aggregation by about 6 h (Fig. 2), an effect that was not observed in *Ddis* [19] and *Pvio* (Fig. 4), although *Pvio dgcAˉ* cells made larger aggregates. Both delayed aggregation and larger aggregates could be caused by a reduced ability of the *dgcAˉ* to initiate primary aggregation centres, a feature that requires further study. In both *Ppal* and *Pvio, dgcAˉ* mutants still formed stalked fruiting bodies, but their stalks were very long and thin. In *Ppal dgcAˉ*, the formation of its typical whorls of secondary sorogens was much delayed and occurred only nearer the top of the primary stalk. This was less obvious in *Pvio* where secondary sorogen formation is already sparse in the wild-type. The spore heads of *Pvio* and *Ppal dgcAˉ* fruiting bodies were very small and their overall sporulation efficiency was 95% reduced for *Pvio* and 87% for *Ppal.* Similar to wild-type, early *Pvio dgcAˉ* sorogens showed prespore differentiation up to the tip (Fig. 6), which makes it more likely that depletion of cells by the very extended stalk formation is the cause of the low number of spores, rather than a direct requirement of c-di-GMP for sporulation. Normal fruiting body morphogenesis was restored in *Pvio dgcAˉ* by complementation with *dgcA* indicating that the defective phenotype was due to loss of c-di-GMP.

### The *Pvio* and *Ppal dgcAˉ* phenotypes resemble their *acaAˉ* phenotypes

In *Ddis*, the effect of c-di-GMP on stalk gene induction and stalk cell differentiation is mediated by the adenylate cyclase ACA acting on PKA, with ACA being strongly activated by c-di-GMP [20]. We recently deleted the three *aca* genes of *Ppal* individually and in combination [40]. *Ppal aca1ˉ* cells made longer and thinner stalks, *aca2ˉ* cells showed delayed secondary sorogen formation and *aca3ˉ* cells formed less aggregation centers. Double *aca1ˉaca2ˉ* and *aca1ˉaca3ˉ* mutants combined the phenotypes of the single knock-outs, but both *aca3ˉaca2ˉ* and *aca1ˉaca3ˉaca2ˉ* mutants also showed > 24 h delayed aggregation, with only few aggregation centres and some small fruiting bodies being formed.

*Ppal dgcAˉ* phenocopies the defects of individual *Ppal acaˉ* mutants, but its aggregation delay is less severe than that of the *aca3ˉaca2ˉ* and *aca1ˉaca3ˉaca2ˉ* mutants. This suggests that also here the effects of c-di-GMP are mediated by ACAs, with ACA1, ACA2 and ACA3 regulating different but overlapping aspects of the developmental program. A mutant in the single *Pvio acaA* gene also made fruiting bodies with longer and thinner stalks [41], but less so than the *Pvio dgcAˉ* mutant, while a double knockout in *Pvio acaA* *a*nd another adenylate cyclase *acrA* could not form stable aggregates. Similar to *Ddis acaA*, the *Ppal* and *Pvio aca* genes are all preferentially expressed in the tip and stalk cells supporting the genetic and biochemical evidence that DgcA and the ACAs act in the same signalling pathway that triggers stalk cell differentiation at the sorogen/slug tip [20].

The aggregation defects of the *Ppal* and *Pvio acaA* or *acaAˉacrAˉ* mutant could be rescued by the PKA agonist 8Br-cAMP {Kawabe, 2023 #8598;Kawabe, 2022 #8597} indicating that like *Ddis* {Harwood, 1992 #1481}, *Pvio* and *Ppal* require PKA activity for aggregation. The relatively mild aggregation deficiencies of the *Pvio* and *Ppal dgcAˉ* cells (Figs. 2 and 4) may indicate that also at this stage c-di-GMP acts to activates AcaA and thereby PKA. However, a role for c-di-GMP, independent of AcaA, cannot be excluded.

### ***Pvio*** stalk formation does not require StlB, AmtC or PhyA

The observation that unlike *Ddis dgcAˉ*, the *Ppal* and *Pvio dgcAˉ* mutants still formed a stalk, led us to consider which other signals might additionally be required for stalk formation. One possibility is DIF-1, produced by the polyketide synthase StlB, which induces differentiation of the stalk-like basal disc cells in *Ddis* [18]. In *Pvio*, which does not form a basal disc, deletion of *stlB* caused stalk abnormalities, but stalks were irregular and thicker than in wild-type *Pvio* [26]. However, the apparent involvement of DIF-1 in *Pvio* stalk formation prompted us to create a *dgcAˉstlBˉ* double mutant. The *dgcAˉstlBˉ* mutant still showed the long thin stalks of the *dgcAˉ* mutant, indicating that DIF-1 does not induce the residual stalk cell differentiation in *Pvio dgcAˉ*.

The interaction of DgcA with ACA merges tip-specific induction of stalk formation with the tip’s role as organiser of morphogenesis, since ACA also partakes in the signalling network that generates the cAMP waves that emerge from the tip to control fruiting body morphogenesis [20, 42, 43]. Before such dedicated signalling mechanisms arose in early evolving Dictyostelia, ambient conditions may have favored stalk formation from the top of the structure. Regulation by two such conditions still acts on stalk cell differentiation in *Ddis*.

One such factor is NH_3_, an inhibitor of stalk cell differentiation, which is abundantly produced by protein degradation in the starving cells. NH_3_ acts on the sensor histidine kinase DhkC to activate the intracellular cAMP phosphodiesterase RegA, thereby preventing PKA activation by ACA [33]. The position of the aerially exposed tip cells, combined with the action of the ammonia transporter AmtC, locally decreases NH_3_ at the tip allowing PKA to be activated and stalk cells to differentiate. Similar to *Ddis dgcAˉ*, *Ddis amtCˉ* mutants show a “slugger” phenotype, because they cannot form the stalk [34, 44]. However, we found that deletion of *amtC* in *Pvio* had no effect on stalk formation, while a *Pvio dgcAˉamtCˉ* double mutant showed the same phenotype as the *dgcAˉ* single mutant (Fig. 8). The role of AmtC in *Ddis* morphogenesis therefore likely evolved only recently, possibly to allow for the stalkless slug migration that is displayed by *Ddis* and some other group 4 species.

A second ambient cue for stalk induction is oxygen availibity [37] which due to its aerial position and narrow shape is likely also highest at the tip. The prolyl 4-hydroxylase PhyA is the only known oxygen sensor in *Ddis*, with *Ddis phyAˉ* cells also showing a “slugger” phenotype and no fruiting body formation [38, 39]. In *Pvio*, deletion of *phyA* had no discernable negative effects on fruiting body formation, while a *dgcAˉphyAˉ* double mutant displayed the long and thin stalk phenotype of the *dgcAˉ* single mutant. It therefore appears that oxygen sensing by *phyA* does not play an evolutionary conserved role in stalk formation.

In short, this study shows that c-di-GMP is a deeply conserved signal for induction of normal stalk cell differentiation in Dictyostelia, but that outside of group 4, it shares this ability with at least one other signal that remains as yet unknown.

## Conclusions

- In contrast to *Ddis*, where c-di-GMP and its synthetic enzyme DgcA are essential for stalk formation, *Ppal* and *Pvio dgcAˉ* mutants still form long and thin stalks.
- *Ppal* and *Pvio dgcAˉ* mutants show delayed aggregation and reduced aggregation centre initiation respectively, indicating that in these species c-di-GMP is also required for early development.
- *Ppal* and *Pvio dgcAˉ* mutants phenocopy their *acaAˉ* mutants, indicating that c-di-GMP induced activation of AcaA, as observed in *Ddis*, is conserved throughout dictyostelid evolution.
- Sporulation efficiency is much reduced in *Ppal* and *Pvio dgcAˉ*, but initial differentiation of prespore cells is normal, suggesting that the extended stalk formation in these mutants depletes the prespore pool.
- Other factors that promote stalk formation in *Ddis* are DIF-1, produced by StlB, low ammonia, facilitated by AmtC, and high oxygen, detected by PhyA. Deletion of *stlB*, *amtC* and *phyA* genes in *Pvio* wild-type and *dgcAˉ* did not reduce stalk formation in either strain.
- The latter observation suggests involvement of another unknown factor in ancestral stalk induction, although overlapping involvement of multiple factors can not be excluded.

## Methods

### Cell culture and development

*Polysphondylium pallidum (Ppal) PN500 (Heterostelium album PN500) and Polysphondylium violaceum (Pvio)*, strain QSvi11 were routinely grown in association with *Klebsiella aerogenes* or *Escherichia coli 281*, respectively, on 1/5th SM agar (Formedium, UK). All strains were obtained from the *Dictyostelium* Stock Center http://dictybase.org/StockCenter/StockCenter.html. For multicellular development, *Pvio* cells were harvested in KK2 (16 mM KH_2_PO_4_ and 4 mM K_2_HPO_4_), washed free from bacteria and spread at 10^6^ cells/cm^2^ on KK2 agar (1.5% agar in KK2). After incubation at 4^o^C overnight, the cells were incubated at 22^o^C until the desired developmental stages had been reached. *Ppal* cells were similarly distributed on NN agar (1.5% agar in 8.8 mM KH_2_PO_4_ and 2.7 mM Na_2_HPO_4_) to induce multicellular development.

### *dgcA* promoter-lacZ constructs and analysis

#### Ppal dgcA promoter-LacZ construct

A 2567 bp fragment ranging from starting − 2507 to + 60 nt relative to the start codon was amplified from *Ppal* gDNA using primer pair PpDGCprF/PpDGCprR (Additional File 1, Table S1), which contain *Xba*I and *Bgl*II restriction sites, respectively. The PCR product was ligated into *Xba*I/*Bgl*II digested vector pDdGal17 [45], yielding vector pPpdgcA-LacZ and transformed into wild-type *Ppal* cells.

#### Pvio dgcA promoter-LacZ construct

The *Pvio dgcA* 2.8 kb promoter was amplified from *Pvio* gDNA using primer pair Pv-dgcA-P51K/Pv-dgcA-P31B that harbour *Kpn*I and *Bam*HI sites respectively. After digestion with *Kpn*I/*Bam*HI, the PCR product was ligated into the *Kpn*I/*Bam*HI digested pDdGal16 [45], yielding vector pPvdgcA-LacZ, which was transformed into wild-type *Pvio*.

#### LacZ staining

The transformed cells were harvested from growth plates, distributed at 10^6^ cells/cm^2^ on nitrocellulose filters supported by NN agar for *Ppal* and dialysis membrane supported by KK2 agar for *Pvio*. Cells were incubated at 22^o^C until the desired developmental stages had been reached. Filters with developing structures were fixed in glutaraldehyde and stained with X-gal as previously described [46].

### Gene disruption constructs and knock-out diagnoses

#### Ppal dgcA knock-out construct

For gene disruption of *Ppal dgcA* (locus tag: PPL_07541, Genbank: EFA79490) two fragments, KOI and KOII, were amplified from *Ppal* PN500 clone 2 genomic DNA using primer pairs DgcAI5’/DgcAI3’ and DgcAII5’/DgcAII3’ (Additional File 1, Table S1) for KOI and KOII, respectively. After digestion of KOI with *Xba*I and *BamH*I and of KOII with *Hind*III and *Xho*I, the fragments were sequentially ligated into *Xba*I/*BamH*I and *Hind*III/*Xho*I digested plasmid pLox-NeoIII [47], creating pPp-dgcA_KO. For transformation, cells harvested from growth plates were incubated for 5 h in HL5 at 2.5 × 10^6^ cells/ml and resuspended in H50 buffer. 90 µl of cells were transformed with 10 µl 0.5 µg/µl linearised pPp-dgcA_KO and 0.5 nanomoles of the flanking primers DgcAI5’ and DgcAII3’ in 1 mm gap cuvettes with two pulses of 0.65 kV/25 µFd at a 5 s interval. Recovery and selection of transformants was performed as described before [48]. Genomic DNA was isolated from G418 resistant clones and screened for homologous recombination with primer pair PpDgcneg5’/ PpDgcneg3’, which amplify a fragment of 0.35 bp in wild-type cells and random integrants and primer pair Dgcpos/G418f which amplify a 1.35 kb fragment only in knock-outs (Additional File 1, Figure S2).

#### Pvio dgcA knock-out construct

To disrupt *Pvio dgcA* (locus tag Pvio_g2456, Genbank KAF2076215), a *dgcA* fragment was amplified from *Pvio* QSvi11 genomic DNA by PCR using primerpair Pv-dgcA-51X and Pv-dgcA-31 K (Table S1) that harbour *Xba*I and *Kpn*I sites respectively. The fragment was cloned into the *Xba*I and *Kpn*I sites of pBluescript SK+. The resulting plasmid was digested with *Eco*RI and *Hind*III and ligated to the actin6-NeoR cassette, which was excised with *Eco*RI/*Hind*III from pLoxNeoII [8]. This yielded vector pPv-dgcA-KO, with the actin6-NeoR cassette flanked by 1.7 and 1.8 kb of *Pvio* gDNA. The vector was linearized with *Sal*I/*Xba*I and transformed into *Pvio* as described previously [26]. Gene knock-out was diagnosed by digesting gDNA of wild-type and transformed clones with *Cla*I and *Bgl*II and analysing the digests by Southern blot, using a ^32^PdATP-labeled *Hind*III/*Sal*I fragment of pPv-dgcA-KO as a probe (Additional File 1, Figure S3).

#### Complementation of Pvio dgcA^−^ with dgcA

A 5.0 kb fragment that contains the 2.8 kb *Pvio dgcA* 5’intergenic region, coding sequence and terminator was amplified from gDNA using Phusion High-Fidelity DNA polymerase (Thermo Fisher) and primers Pv-dgcA-P51K/Pv-dgcA-31 K that harbour a *Kpn*I restriction site. The PCR product was digested with *Kpn*I/*Sal*I, using an internal *Sal*I site near the end of terminator, and cloned into pBluescript SK + for sequence validation. The validated plasmid was digested with *Kpn*I and *Sma*I and the insert was cloned into *Kpn*I and *Sma*I digested vector pHygTm(+)/pG7 vector (http://dictybase.org/db/cgi-bin/dictyBase/SC/plasmid_details.pl?id=453), which contains the hygromycin resistance cassette, yielding vector pPv-DgcA-Exp, which was introduced into *Pvio dgcAˉ* cells. To select transformants, the cells were incubated in 10 ml KK2, containing autoclaved *Klebsiella aerogenes* (final OD_600_ = 4.2), 10% HL5 and 30 µg/ml hygromycin, incubated in 9 cm dishes for 48 h, and then distributed with *E.coli* 281 on 1/5th SM agar, containing 30 µg/ml of hygromycin.

#### Disruption of stlB in the Pvio dgcAˉ mutant

To remove the NeoR cassette, *Pvio dgcAˉ* cells were transformed with pA15NLS.Cre [49] for transient expression of Cre-recombinase and G418 sensitive clones were selected. One clone, checked for having retained the *dgcAˉ* phenotype was transformed with a previously constructed vector pPvStlB-KO [26]. Genomic DNAs from G418 resistant clones were isolated and tested by PCR for knock-out of stlB using primer pairs Pv_stlB_NegF/Pv_stlB_NegR and Pv_stlB_Pos5’/cas1 (Additional File 1, Figure S4).

#### Pvio amtC knock-out construct

To disrupt *amtC *(Pvio_g4319, KAF2074385), two fragments, KO1 and KO2, of 1082 and 1019 bp, respectively, were amplified from P*vio* QSvi11 gDNA using primer pairs Pv-amtC-51 K/Pv-amtC-31 C Pv-amtC-52B/Pv-amtC-32X, which contain *Kpn*I/*Cla*I and *Bam*HI/*Xba*I sequences, respectively. Fragment KO1 was digested with *Kpn*I/*Cla*I and inserted into *Kpn*I/*Cla*I digested vector pLox-NeoIII. Next, fragment KO2 was digested with *Bam*HI/*Xba*I and inserted into the *Bam*HI/*Xba*I sites of the new vector, generating vector pPv-amtC-KO. This vector was transformed into both *Pvio* wild-type cells and in the G418 sensitive *dgcA*ˉ cells, generated as described above. Homologous recombination was assessed in G418 resistant clones by PCR using primer pair Pv-amtC-54/Pv-amtC-34 (Additional File 1, Figure S5).

#### Pvio phyA knock-out construct

The *Pvio* genome contig_1692, which harboured *phyA* (locus tag Pvio_g10567, Genbank KAF2068107), was just 2200 nt long and apart from the coding region, contained only 284 and 981 nt 5’ and 3’ intergenic sequence, respectively. To obtain a longer stretch of 5’ UTR, inverse PCR from *EcoR*I digested and religated *Pvio* gDNA was performed, using primers Pv-PhyA-51B and Pv-phyA-31S, which harbour *Bam*HI and *Sac*I sites respectively and are complementary to sequences within the *phyA* coding region. A ~ 5 kb fragment was amplified, which was digested with *Bam*HI and *Sac*I and cloned into pLoxNeoI∆EcoRI, yielding pPvio-phyA-KO. The fragment was also cloned into pBluescript SK + for sequence validation, which revealed a *Cla*I site at -678 from the start ATG. The pPvio-phyA-KO vector was digested with *Cla*I and *EcoR*I yielding the entire pLoxNeoI∆EcoRI sequence flanked by 817 bp of 5’ PhyA sequence and 1137 bp of 3’ phyA sequence (Additional File 1, Figure S6) and transformed into wild-type and *dgcA*ˉ cells. Homologous recombination was assessed in G418 resistant clones by PCR using primer pairs Pv-phyA-52 K /Pv-phyA-32 H (Table S1).

All gene disruption mutants and plasmid constructs described above have been submitted to the Dicty Stock Center http://dictybase.org/StockCenter/StockCenter.html.

### Staining with spore antibodies

Developed structures on dialysis membrane were fixed for 15 min in ice-cold methanol, washed with PBS (0.8% NaCl in 10 mM Na/K phosphate, pH 7.4), supplemented with 5% BSA and incubated for 16 h at 4 °C with a 1:1000 dilution of pre-absorbed anti-spore antibody in PBS/BSA [27]. After three washes with PBS, structures were incubated with 1:100 diluted FITC conjugated goat-anti-rabbit-IgG for 4 h at room temperature. Membranes with structures were mounted onto standard microscope slides for fluorescence microscopy and imaged using a Leica DMLB2 fluorescence microscope.

### RNAseq of *a P. violaceum* developmental time course

*Polysphondylium violaceum (Pvio)*, strain QSvi11 were grown and developed into multicellular structures as described above. Structures from 2 × 10^8^ cells were harvested in KK2 at 0, 4, 16, 20 and 24 hours of development and collected by centrifugation at 2,000 x g. Multicellular structures were dissociated by passing 15x through a 23G needle in RLT buffer (Qiagen). Total RNA was extracted using the RNAasy Midi Kit (Qiagen) and quantified using a UV/Vis spectrophotometer. cDNA libraries were prepared using the Truseq Stranded mRNA Library Prep Kit (Illumina, USA). 75-bp paired-end reads were sequenced with Illumina NextSeq 500 at the Tayside Centre for Genomic Analysis (https://tcga.org.uk) in two independent runs. The sequence data are archived in the European Nucleotide Archive as Project PRJEB59611. To obtain developmental transcription profiles, the software package RSEM v1.3.1 [50] was used to estimate transcript abundances, with the Bowtie2 aligner for mapping RNA-Seq reads to gene features. We used the “--estimate-rspd” option in RSEM to estimate read start position distributions (RSPD), which is expected to facilitate more accurate abundance estimates for 3’ biased reads produced from oligo-dT primed libraries [50]. The expression data in Transcripts Per Million (TPM) are listed in Supplementary_ Dataset_File1_Pvio_RNAseq.xlsx.

### Electronic supplementary material

Below is the link to the electronic supplementary material.

Supplementary Material 1

Supplementary Material 2

## Data Availability

*P. violaceum* developmental RNAseq data are archived in the European Nucleotide Archive https://www.ebi.ac.uk/ena/browser/view/PRJEB59611 as Project PRJEB59611.
