# Supplementary material for: Emerging roles for diguanylate cyclase during the evolution of soma in dictyostelia
Source: BMC Ecol Evol. 2023 Oct 6;23:60. doi: 10.1186/s12862-023-02169-z (PMC10559540; doi:10.1186/s12862-023-02169-z)
Supplement: Supplementary file 2 — Supplementary Material 2 [file 12862_2023_2169_MOESM2_ESM.docx]

**ADDITIONAL FILE 1**

**Supplementary figures S1-S7, supplementary table S1**

**
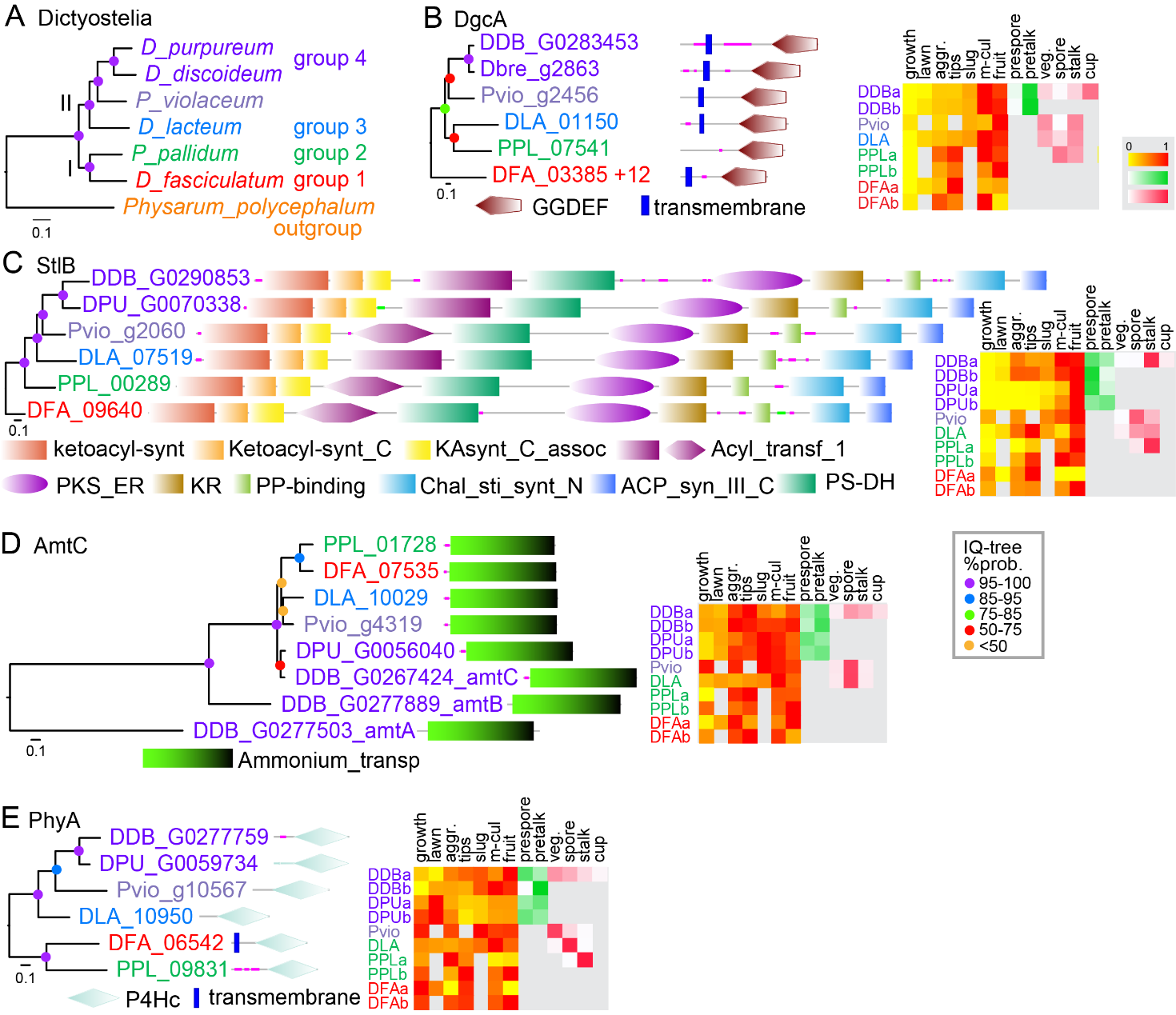
**

**Figure S1. Conservation of *dgcA*, *stlB*, *amtC* and *phyA* genes across Dictyostelia**

A. *Dictyostelium* phylogeny based on 47 concatenated proteins from sequenced genomes [1] shows subdivision into two branches (I and II), that each contain two major groups. *P. violaceum* resides in a minor sister clade to group 4.

B_E. DgcA, StlB, AmtC and PhyA protein sequences were identified by BLASTp from proteomes of the species shown in the phylogeny and for DgcA also the *D. brefeldianum* proteome (Schilde and Schaap, unpublished results). *D.fasciculatum* harbours 13 DgcA proteins, only the least diverged one is shown. To confirm orthology, the highest scoring BLAST hits for all proteins were aligned, phylogenetic relationships were inferred using IQ-tree [2] and conservation of functional domains was analysed by SMART [3]. To compare developmental regulation and cell-type specificity of the *dgcA*, *stlB*, *amtC* and *phyA* genes, normalized read counts were retrieved from published [4-7] and recent (Supplementary_Dataset_File1_Pvio_RNAseq.xlsx) RNAseq experiments. Data are shown as heat maps that represent the fraction of the maximum transcript number for the developmental profiles and the fraction of the summed number for the cell types.


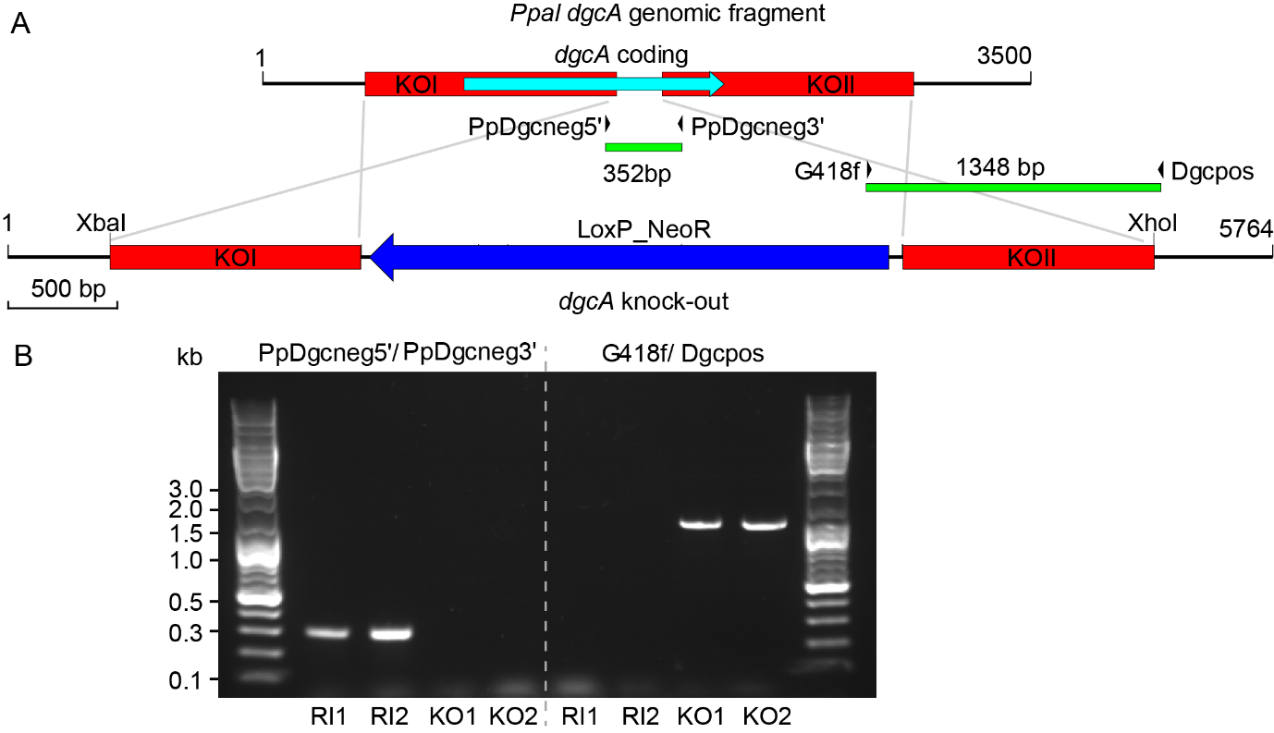


**Figure S2. Disruption of *P. pallidum dgcA***

*A. Knock-out schematic. Ppal* *dgcA* genomic fragment before (top) and after (bottom) homologous recombination with the *dgcA* knock-out construct. The locations of the primers used for knock-out diagnosis are indicated.

*B. Diagnosis.* Amplification of fragments from genomic DNAs of two knock-out (KO) and two random integrant (RI) clones with the indicated primer pairs.


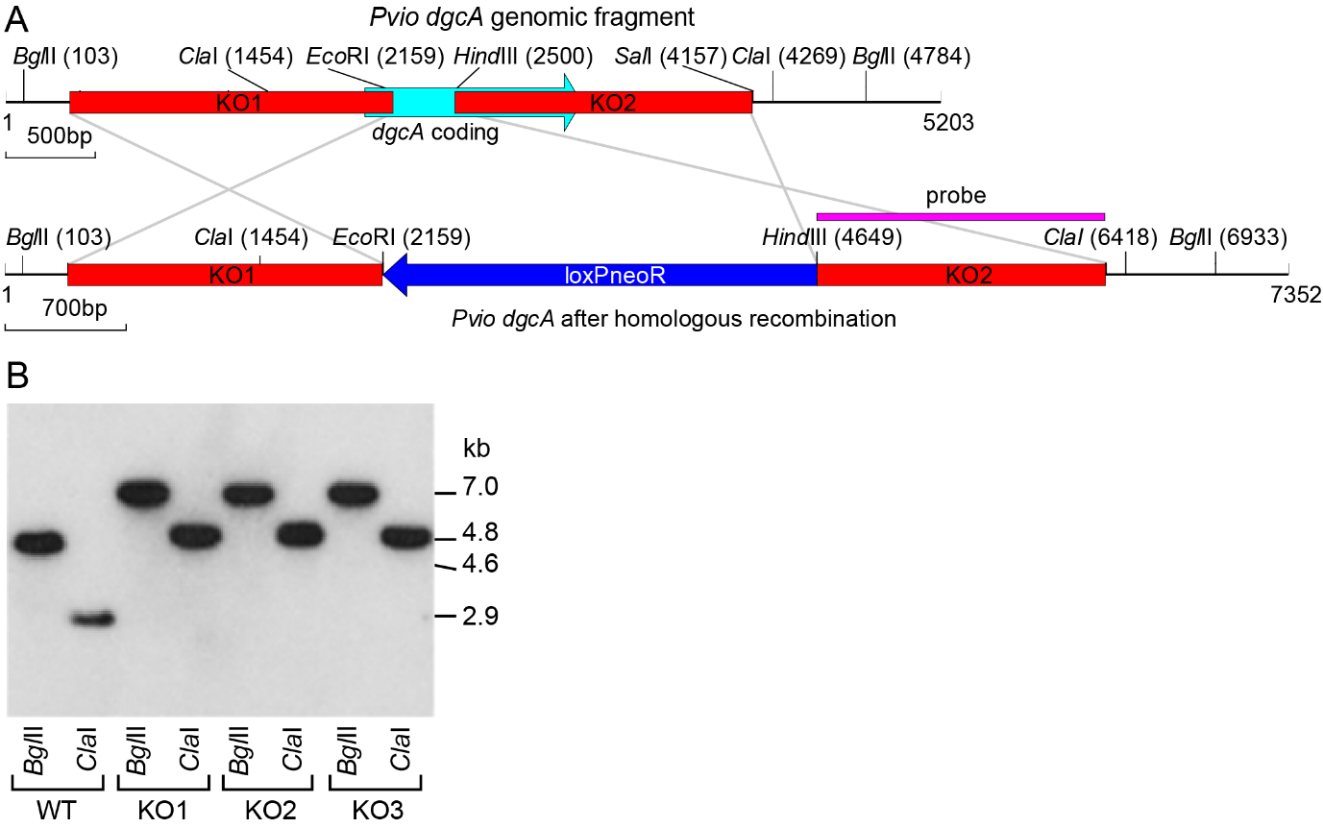


**Figure S3. Disruption of *P.violaceum dgcA***

*A. Knock-out schematic.* Annotated restriction map of a *Pvio dgcA* genomic fragment before (top) and after (bottom) homologous recombination with the knock-out contruct. The location of the probe used for knock-out diagnosis is indicated (bottom).

*B. Diagnosis.* Genomic DNAs of 3 transformants (KO1 to 3) and untransformed cells (WT) were digested with *Bgl*II *or Cla*I and Southern blots were probed with the ^32^P-dATP-labeled fragment that is indicated in A. The detected band in transformants were about 2 kb larger than those in wild type as expected after homologous recombination with the knock-out construct. The full autoradiogram of the Southern blot is shown in figure S7.


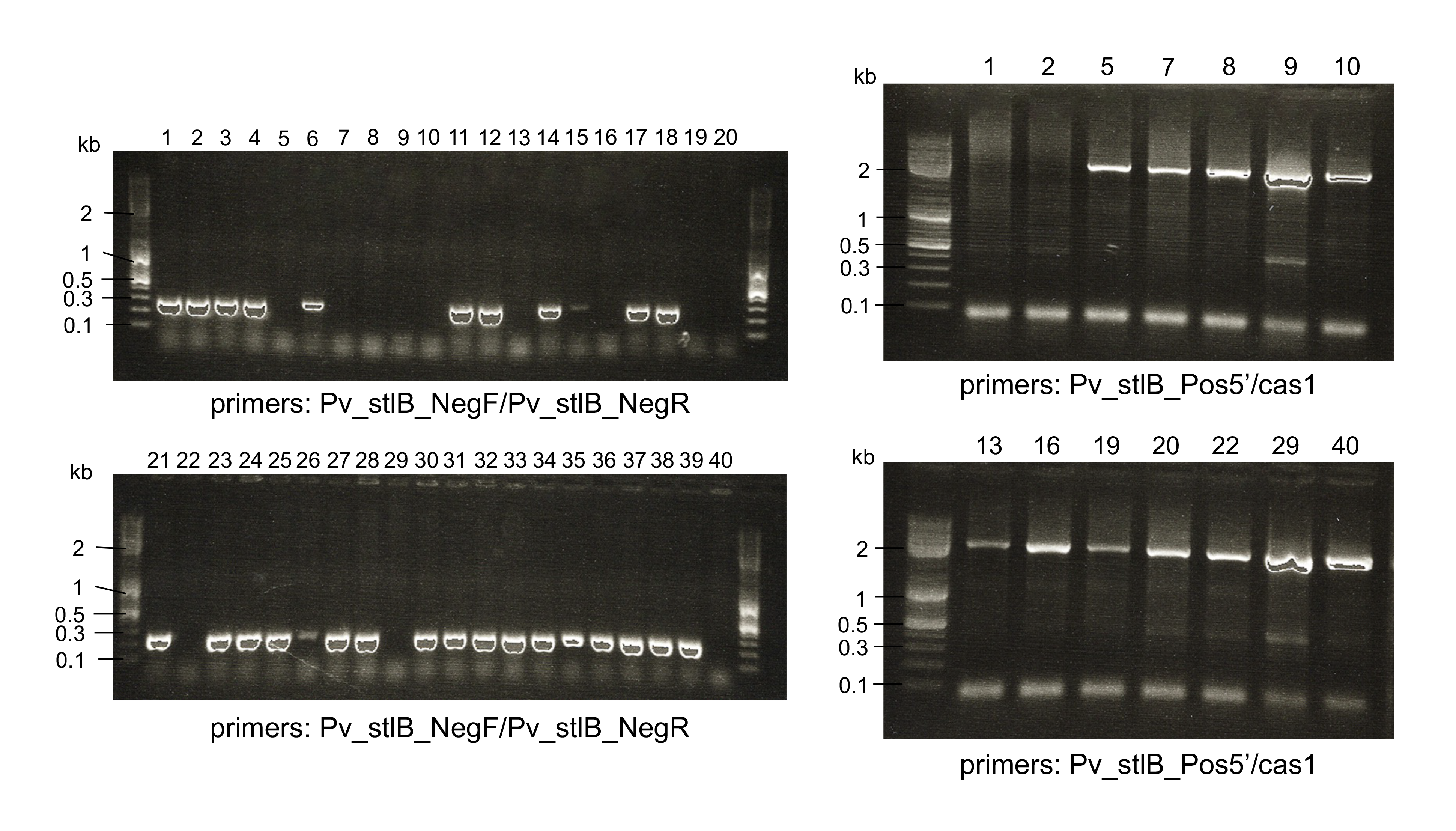


**Figure S4. Disruption of *stlB* in *Pvio dgcAˉ***

A G418 sensitive *dgcAˉ* clone (see Methods) was transformed with a pPvStlB knock-out vector [8] and gDNAs were isolated from transformed G418 resistant clones. Primer pair Pv_stlB_NegF/Pv_stlB_NegR amplifies a 286 bp in WT or *dgcAˉ* only, while primer pair Pv_stlB_Pos5’/cas1 amplifies a 2 kb fragment in *stlB* knock-outs See figure S2 [8] for a schematic. Two likely RI clones (1 and 2) and 5 likely KO clones (5,7-10) were identified by amplification with Pv_stlB_NegF/Pv_stlB_NegR and proved to be correct after amplification with Pv_stlB_Pos5’/cas1.

**
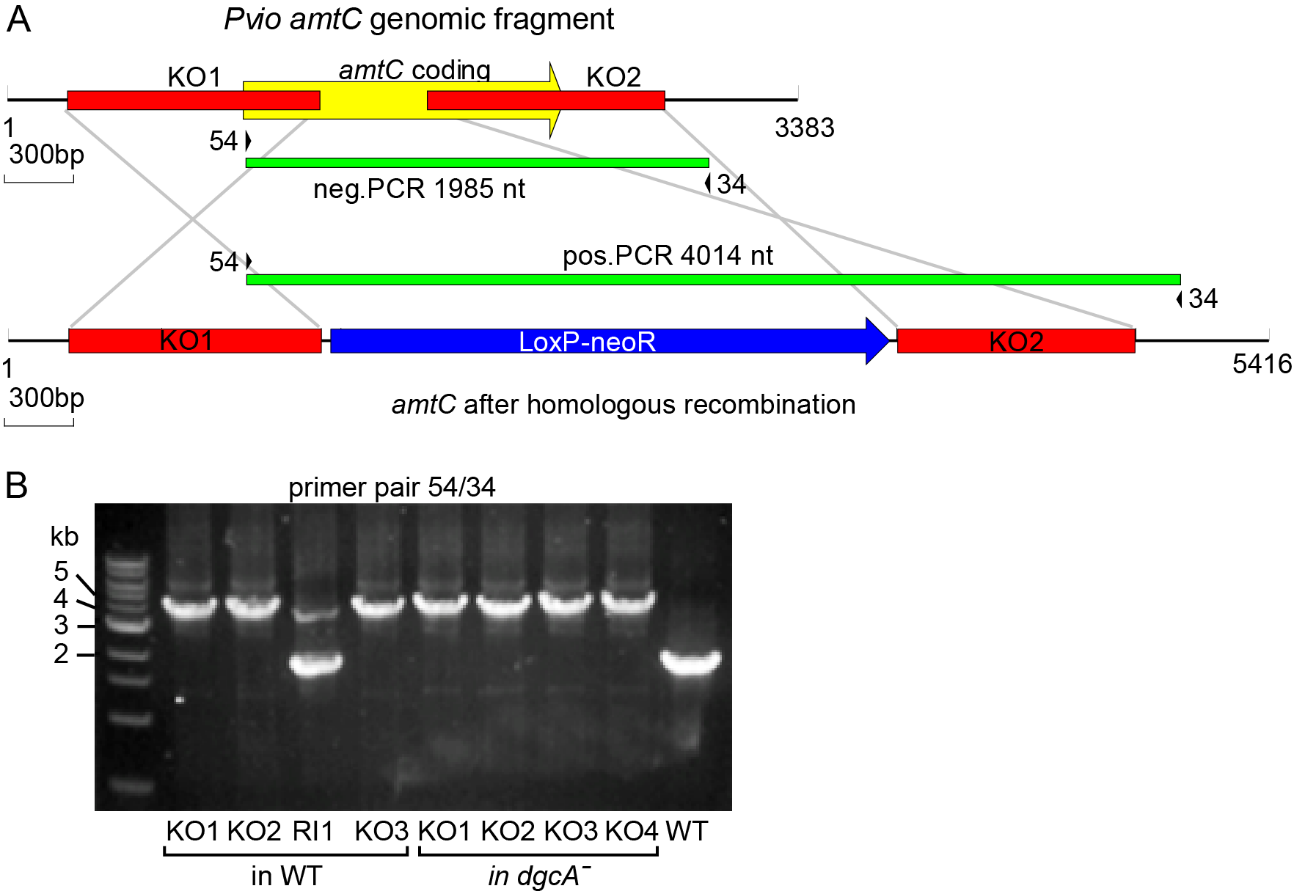
**

**Figure S5. Disruption of *Pvio amtC***

*A. Knock-out schematic. Pvio* *amtC* genomic fragment before and after homologous recombination with the location of the primers used for knock-out diagnosis.

*B.* *Diagnosis.* Both *Pvio* wild-type and *dgcA*ˉ cells were transformed with the pPv-amtC-KO vector and gDNA were isolated from transformed clones. Primer pair Pv-amtC-54/Pv-amtC-34 amplifies a 2.0 kb fragment in WT and a 4.0 kb fragment in knock-outs.


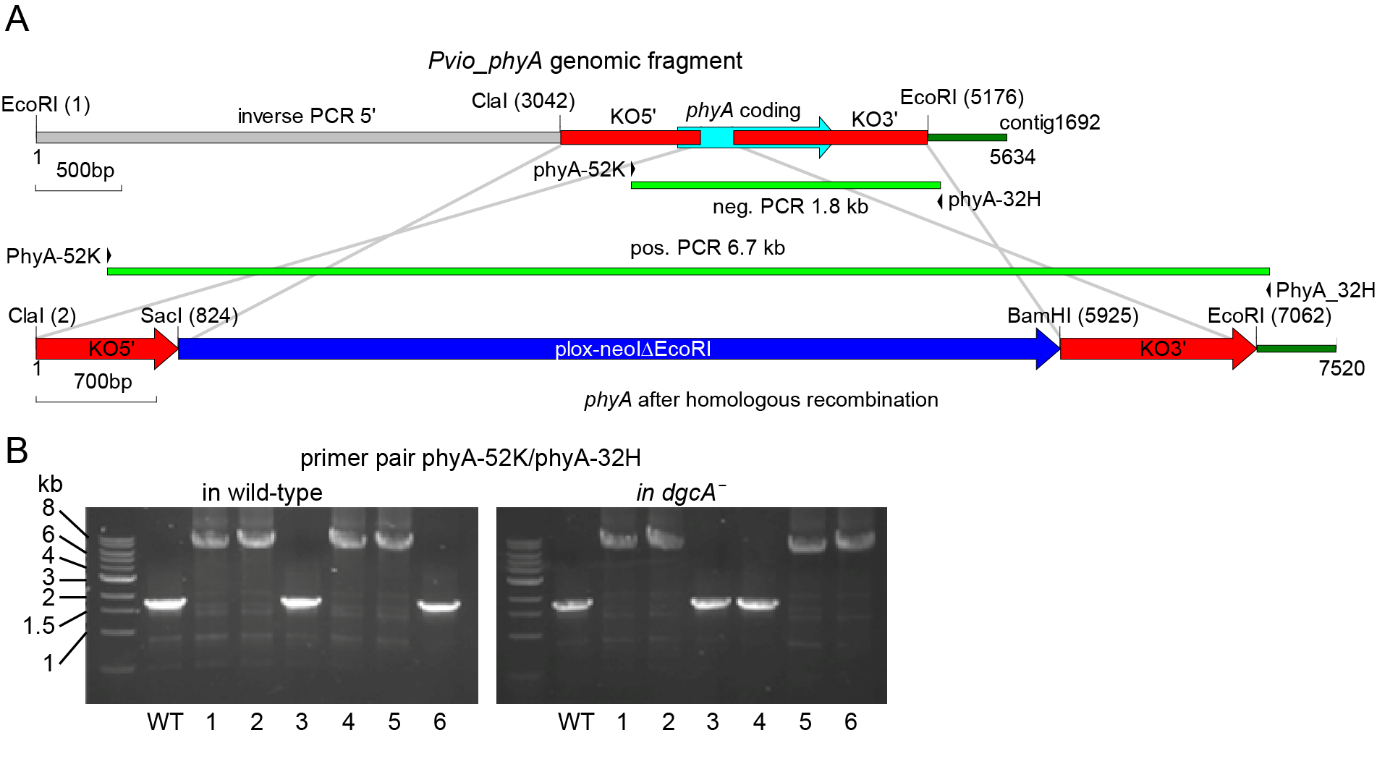


**Figure S6. Disruption of *Pvio phyA***

*A. Knock-out schematic. Pvio PhyA* resided on a 2.2 kb contig. A longer genomic segment was amplified by inverse PCR from *EcoR*I digested, religated *Pvio* gDNA and cloned into pLoxNeoIΔEcoRI, which was linearized with *Cla*I and *EcoRI* and transformed into *Pvio* wild-type and *dgcAˉ* cells.

B. *Diagnosis. gDNAs from transformed* wild-type and *dgcA*ˉ cells amplified with primer pair phyA-52K/phyA-32H, which generates a 1.8 kb fragment in WT and random integrants and a 6.7 kb fragment in knock-outs. The gel images show that 4 of 6 clones were knock-outs in both cases.


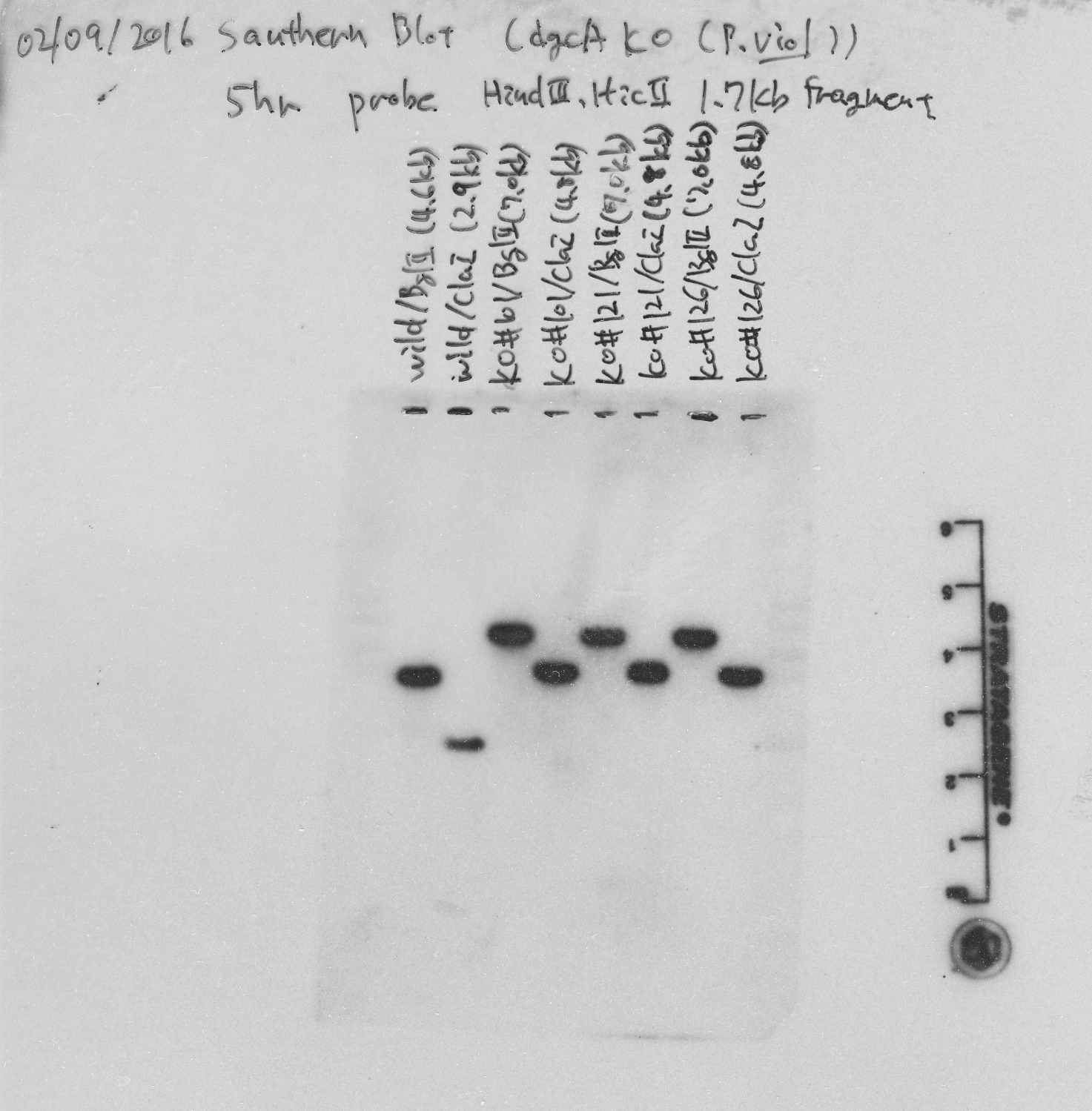


**Figure S7. Full autoradiogram of the Southern blot of figure S3**

**Table S1. Oligonucleotide primers used in this work**

| **Name** | **Restrict. Site** | **Sequence (restriction site in bold)** |
| --- | --- | --- |
| PpDgcAI5’ | XbaI | GAT**TCTAGA**GGTTTAATTGCAGACATCAC |
| PpDgcAI3’ | BamHI | GAT**GGATCC**AGTGAGTGGATCGTGTG |
| PpDgcAII5’ | HindIII | GAT**AAGCTT**TCTGGCGACGAGTTTCTC |
| PpDgcAII3’ | XhoI | GAT**CTCGAG**GTGGACATGCCCGCTTATA |
| PpDgcneg5’ |  | GAGTCGGAATTGAAACGTCTC |
| PpDgcneg3’ |  | GCGGCGACTCCATTCAAGTAGT |
| PpDgcpos |  | GTTTGGTATTTTATACAGGTGATC |
| G418f |  | GGGCAAATCTGTAATTTTCAG |
| PpDGCprF | XbaI | AA**TCTAGA**ATCTAATAGAAAGATATCTAACACAACCAC |
| PpDGCprR | BglII | AA**AGATCT**GATTTGAAATTTATATTGTTGGCTGGTTGTTG |
| Pv-dgcA-51X | XbaI | GC**TCTAGA**CCCTCACATCTGTAATACGG |
| Pv-dgcA-31K | KpnI | GG**GGTACC**GCTTTTGTTGTCGATAGACACT |
| Pv-dgcA-P51K | KpnI | GG**GGTACC**TAGTTGTTGGTGTTCCTCTTC |
| Pv-dgcA-P31B | BamHI | CG**GGATCC**AATGTTCAACCACTTTGGTTTC |
| Pv_stlB_NegF |  | CAAGCAATATGGACACTGCTCAAG |
| Pv_stlB_NegR |  | GACTTACTGGTCTTAAAGGTGGAG |
| Pv_stlB_Pos5' |  | TACTCTTACCAATGAAATGCACTC |
| cas1 |  | GGGCAAATCTGTAATTTTCAG |
| Pv-amtC-51K | KpnI | GG**GGTACC**CAAATGAAGAAAAAGCCT |
| Pv-amtC-31C | ClaI | CC**ATCGAT**ACCAAACGCAAAGGAATGT |
| Pv-amtC-52B | BamHI | CG**GGATCC**GCATGACCAAAAACAGAGTG |
| Pv-amtC-32X | XbaI | GC**TCTAGA**AAGAGAGTGTGAAGGTGA |
| Pv-amtC-54 |  | CATCATCGTCAGATTCAACA |
| Pv-amtC-34 |  | AGCTATCTTCTTCGAATTCC |
| Pv-phyA-51B | BamHI | CG**GGATCC**GAACAAAGGTGCTGATAGATGG |
| Pv-phyA-31S | SalI | GC**GAGCTC**AAGGTACTCTTGACTGATGATG |
| Pv-PhyA-52K |  | CGGGTACCTTATCATTGGCTCTGTCGG |
| Pv-PhyA-32H |  | CCCAAGCTTATAGGGTGAAAAGAGTGC |

**SUPPLEMENTAL REFERENCES**

1. Singh R, Schilde C, Schaap P: A core phylogeny of Dictyostelia inferred from genomes representative of the eight major and minor taxonomic divisions of the group. *BMC Evol Biol* 2016, 16(1):251.

2. Trifinopoulos J, Nguyen L-T, von Haeseler A, Minh BQ: W-IQ-TREE: a fast online phylogenetic tool for maximum likelihood analysis. *Nucleic Acids Research* 2016, 44(W1):W232-W235.

3. Schultz J, Milpetz F, Bork P, Ponting CP: SMART, a simple modular architecture research tool: identification of signaling domains. *Proc Natl Acad Sci USA* 1998, 95(11):5857-5864.

4. Parikh A, Miranda ER, Katoh-Kurasawa M, Fuller D, Rot G, Zagar L, Curk T, Sucgang R, Chen R, Zupan B *et al*: Conserved developmental transcriptomes in evolutionarily divergent species. *Genome Biol* 2010, 11(3):R35.

5. Kin K, Forbes G, Cassidy A, Schaap P: Cell-type specific RNA-Seq reveals novel roles and regulatory programs for terminally differentiated Dictyostelium cells. *BMC Genomics* 2018, 19(1):764.

6. Gloeckner G, Lawal HM, Felder M, Singh R, Singer G, Weijer CJ, Schaap P: The multicellularity genes of dictyostelid social amoebas. *Nature communications* 2016, 7:12085.

7. Forbes G, Chen ZH, Kin K, Lawal HM, Schilde C, Yamada Y, Schaap P: Phylogeny-wide conservation and change in developmental expression, cell-type specificity and functional domains of the transcriptional regulators of social amoebas. *BMC Genomics* 2019, 20(1):890.

8. Narita TB, Kawabe Y, Kin K, Gibbs RA, Kuspa A, Muzny DM, Richards S, Strassmann JE, Sucgang R, Worley KC *et al*: Loss of the Polyketide Synthase StlB Results in Stalk Cell Overproduction in Polysphondylium violaceum. *Genome Biol Evol* 2020, 12(5):674-683.
